# Implementing guidelines in nursing homes: a systematic review

**DOI:** 10.1186/s12913-016-1550-z

**Published:** 2016-07-25

**Authors:** Heinz Diehl, Birgitte Graverholt, Birgitte Espehaug, Hans Lund

**Affiliations:** 1 Centre for Evidence-Based Practice, Bergen University College, Inndalsveien 58, 5063 Bergen, Norway; 2 Department of Sports Science and Biomechanics, University of Southern Denmark, Campusvej 55, 5230 Odense, Denmark

**Keywords:** Nursing homes, Guideline implementation, Knowledge translation, Systematic review

## Abstract

**Background:**

Research on guideline implementation strategies has mostly been conducted in settings which differ significantly from a nursing home setting and its transferability to the nursing home setting is therefore limited. The objective of this study was to systematically review the effects of interventions to improve the implementation of guidelines in nursing homes.

**Methods:**

A systematic literature search was conducted in the Cochrane Library, CINAHL, Embase, MEDLINE, DARE, HTA, CENTRAL, SveMed + and ISI Web of Science from their inception until August 2015. Reference screening and a citation search were performed. Studies were eligible if they evaluated any type of guideline implementation strategy in a nursing home setting. Eligible study designs were systematic reviews, randomised controlled trials, non-randomised controlled trials, controlled before-after studies and interrupted-time-series studies. The EPOC risk of bias tool was used to evaluate the risk of bias in the included studies. The overall quality of the evidence was rated using GRADE.

**Results:**

Five cluster-randomised controlled trials met the inclusion criteria, evaluating a total of six different multifaceted implementation strategies. One study reported a small statistically significant effect on professional practice, and two studies demonstrated small to moderate statistically significant effects on patient outcome. The overall quality of the evidence for all comparisons was low or very low using GRADE.

**Conclusions:**

Little is known about how to improve the implementation of guidelines in nursing homes, and the evidence to support or discourage particular interventions is inconclusive. More implementation research is needed to ensure high quality of care in nursing homes.

**Protocol registration:**

PROSPERO 2014:CRD42014007664

**Electronic supplementary material:**

The online version of this article (doi:10.1186/s12913-016-1550-z) contains supplementary material, which is available to authorized users.

## Background

The number of older people is increasing rapidly in both absolute and relative terms, and the number of people in need of long-term care will increase as a consequence [1]. As a result, the use and societal expense of nursing home care will grow strongly [2]. The most complex care needs are often found in the frail nursing home population, attributed to high levels of disability and the presence of multiple chronic diseases [3]. However, quality of care in nursing homes is of ongoing concern, notably the shortage of implementing high quality evidence into daily care [4, 5].

Clinical practice guidelines (guidelines) provide healthcare personnel with decision support based on the best evidence available in order to improve quality of care and to reduce unwarranted variation in healthcare delivery [6, 7]. Although guideline dissemination is the first step in moving from recommendations to implementation, it is rarely sufficient. An effective implementation strategy is crucial to ensure the use of guidelines in daily practice [8].

Various international reviews have evaluated the effects of guideline implementation strategies on professional practice and patient outcome [9–18]. In addition, a large scoping review examined the extent of knowledge translation studies in relation to older adults [19]. Most studies in these reviews were, however, conducted in acute care, outpatient and primary care settings other than nursing homes. These settings differ from nursing homes in several important factors like the skill-mix, the environment, the case mix and the availability of human and financial resources [19, 20]. Such factors are shown to play an important role in the translation of evidence into practice [21, 22], thus implementation strategies from other settings will be limited in how they can be transferred to the nursing home setting.

This highlights the need for knowledge about evidence-informed implementation strategies on the successful uptake of guidelines in nursing homes. Nursing home providers could benefit from improved understanding on how to enhance guideline implementation. In turn, society could benefit from reduced healthcare costs. And most important, improved quality of care and reduced unwarranted variation in healthcare delivery could result in a better life for nursing home residents. The aim of this study was, therefore, to conduct a systematic review to evaluate the effects of guideline implementation strategies on professional practice and patient outcome in nursing homes. The research question is *“What are the effects of interventions to improve the implementation of guidelines on professional practice and patient outcomes in nursing homes?”*

## Methods

A study protocol describing the details of this review was developed in advance and is available in the International Prospective Register of Systematic Reviews (PROSPERO) (registration number CRD42014007664) [23].

### Eligibility criteria

We considered studies for inclusion if they involved healthcare personnel working in a nursing home providing high-level care, evaluated any type of guideline implementation strategy, compared to any other type of guideline implementation strategy or compared to usual care. The primary outcomes of interest were objective measures of professional practice or patient outcome. Secondary outcomes were subjective outcome measures, for example a change in knowledge, attitudes or the residents’ satisfaction. Studies only reporting secondary outcomes were excluded. Study designs to be included were systematic reviews, randomised controlled trials, non-randomised controlled trials, controlled before-after studies and interrupted-time-series studies with at least three measure points before and after the intervention and a clearly defined entry point. No language, geographical or publication date restrictions were imposed.

The guidelines subject to implementation were required to be based on a review of the literature, their recommendations had to be tied to the findings of the literature search and they had to be publicly available [24]. To facilitate replicability and proper data synthesis, the intervention had to be clearly described.

### Information sources and search

From their inception until August 2015, we searched the electronic databases CINAHL, Embase, MEDLINE, SveMed+, ISI Web of Science, the Database of Abstracts of Reviews of Effects (DARE), the Health Technology Assessment Database (HTA), the Cochrane Database of Systematic Reviews (CDSR) and the Cochrane Central Register of Controlled Trials (CENTRAL). Additionally, we searched the grey literature in ClinicalTrials, OpenGrey and PROSPERO, hand searched the references of the included studies, performed a citation search based on the included studies and screened systematic reviews published by the Cochrane Effective Practice and Organisation of Care (EPOC) review group. No language or document format restrictions were imposed. Furthermore, we screened the included studies on additional search terms not present in our current search strategy to overcome a potential indexing flaw in knowledge translation studies [19]. It was planned to re-run the current search strategies with the new search terms applied, and to match up the new results against the present search results. We used keywords and subject headings where appropriate based on the nursing home setting and the intervention when developing the search strategy. The complete search strategy is available in Additional file 1.

### Study selection and quality assessment

Two authors (HD, BG) independently reviewed titles and abstracts, retrieved possibly relevant articles in full-text and assessed them for inclusion in line with the eligibility criteria. A weighted kappa of the screening results of the first 200 references was calculated to assess if both reviewers’ understanding of the inclusion and exclusion criteria was similar enough to be able to proceed with the screening or if further clarification was needed [25]. We resolved disagreement by discussion and consensus. Two reviewers (HD, BG) independently assessed the risk of bias in the included studies using the EPOC risk of bias tool [26]. We resolved disagreement by discussion and consensus or by consulting a third reviewer (HL).

### Data abstraction

One author (HD) extracted information from the included studies using a customised EPOC data abstraction form [26] (Additional file 2). A second author (BG) checked the results. We resolved any disagreement by discussion and consensus. When additional information was needed, we contacted the study authors by email. We extracted the following information from each included study: full reference, study objectives, participating personnel and residents, characteristics of the intervention and control intervention, outcome measures, study design and the results. To classify intervention components, we used the EPOC taxonomy of interventions [27].

### Data synthesis

Due to heterogeneity in interventions and outcomes of the included studies, a meta-analysis was not possible. Instead, we performed a narrative synthesis of the results and summarised the effectiveness in the categories *professional practice* and *patient outcome.* When possible, we recalculated effect estimates using the statistical software R version 3.1.2 [28]. We calculated risk ratio (95 % CI) for dichotomous data and mean difference (95 % CI) for continuous data. The effect estimates were corrected for clustering. We used the “Grading of Recommendations, Assessment, Development and Evaluation” (GRADE) approach to rate the overall quality of the evidence for each outcome as high, moderate, low or very low [29].

## Results

### Study selection

**Fig. 1:**
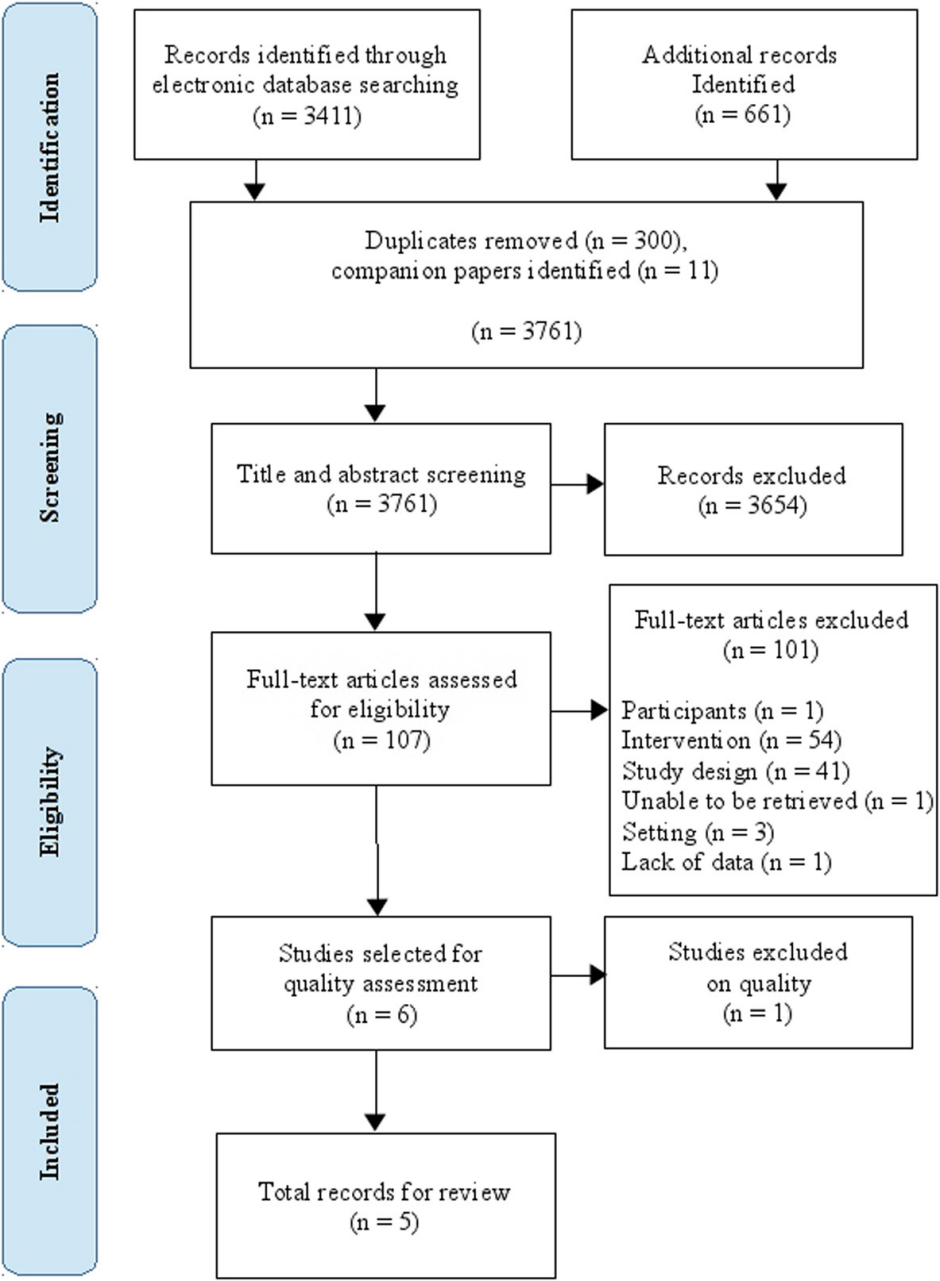
Search and study retrieval process

The literature search yielded 3761 individual articles. Interrater agreement based on the screening results of the first 200 references was strong (k = 0.81). We retrieved 107 articles in full-text, and six met the inclusion criteria. One article was excluded after risk of bias assessment due to severe attrition bias [30]. This judgement was made because non-participation in the intervention activities was extremely high. We finally included five trials [31–36]. One study was reported in two complementary articles [33, 34]. Figure 1 shows the selection process. A table of excluded studies is provided in Additional file 3. No new search terms were identified.

### Study characteristics

The five included studies were cluster-randomised controlled trials using different multifaceted implementation strategies based on education meetings and the distribution of educational material. None of the included studies reported on the secondary outcomes of this review. A total of 9750 residents from 184 nursing homes with a mean age of 83 years participated. One study did not report the number of included residents [36]. Study length ranged from 6 to 23 months. The characteristics of the included studies are summarised in Table 1. Table 2 shows a detailed description of the interventions. Additional files 4 and 5 provide a summary of findings. All results are corrected for cluster design where appropriate.

**Table 1:** Characteristics of included studies

Study, nationality and design	Participating providers (Level of training)	Participating residents (Clusters, number)	Outcome (relevant for this systematic review)	Outcome measurement	Outcome measurement frequency/period	Length of post-intervention follow-up
De Visschere et al. (2012) [31], Belgium. Cluster-randomised controlled trial.	Nurses, nurse aids. Level of training not stated.	12 nursing homes (*N =* 297). Nursing home residents, mean age 84 years, high degree of physical disability and cognitive impairment.	*Patient outcome*: oral hygiene level of the participating residents: dental plaque, denture plaque, tongue plaque (primary outcomes).	Dental plaque: Silnes and Löe plaque index. Denture plaque: Augsburger and Elahi Methylene-blue test. Tongue plaque: Winkel tongue coating index. Tests carried out by trained external examiners.	Measured once after the 6 months intervention period.	-
Köpke et al. (2012) [32], Germany. Cluster-randomised controlled trial.	Nurses with three years of vocational training, certified nurse assistants with 1 year vocational training, untrained nurse assistants.	36 nursing homes (*N =* 3670). Nursing home residents, mean age 85.5 years, high degree of physical disability and cognitive impairment.	*Professional practice*: the number of residents with physical restraints after 6 months (primary outcome). Restraint use at 3 months (secondary outcome).	Unannounced observation by blinded investigators on three different occasions during one day.	Measured after 3 and 6 months during the 6 months intervention period.	-
Tjia et al. (2015) [36], USA. Cluster-randomised controlled trial.	Nurses, certified nurse assistants, physicians, nursing home leaders. Level of training not stated.	42 nursing homes (*N =* unknown).	*Professional practice*: Facility-level change in atypical antipsychotic prescribing rates (primary outcome).	Screening of pharmacy dispensing data. Not stated who assessed the data.	Measured monthly during the 12 months intervention period.	-
Van Gaal (2011a/b) [33, 34], Netherlands. Cluster-randomised controlled trial.	Nurses. Level of training not stated.	10 wards from 6 nursing homes (*N =* 392). Nursing home residents, mean age 78 years, half of them physically impaired. No cognitive impairment.	*Patient outcome* (Part I): Incidence of adverse events: pressure ulcer, urinary tract infections and falls (primary outcome). *Professional practice* (Part II): adequate care given to nursing home residents at risk of adverse events (secondary outcome).	Primary outcome: chart review and inspection of patient’s skin by independent research assistants. Secondary outcome: chart review and patient observation by independent research assistants.	Primary outcomes: measured weekly during post-intervention follow up. Secondary outcomes: measured weekly during post-intervention follow-up. Three additional observational visits on every ward. No measurement during the 14 months intervention period.	9 months.
Ward et al. (2010) [35], Australia. Cluster-randomised controlled trial.	Nursing home staff including physicians. Level of training not stated.	88 nursing homes (*N =* 5391). Nursing home residents, mean age 85.5 years, about 70 % able to stand or walk with or without assistance, 21 % received dementia-specific care.	*Professional practice*: use of vitamin D supplements, use of hip protectors (primary outcomes). *Patient outcome*: change in fall rates, residents with a fractured neck of femur (primary outcomes).	Chart review by nursing home staff.	Monthly during the 17 months intervention period.	-

**Table 2:** Detailed description of interventions

Study	Objective(s) / Intervention	Target population	Comparator	KT activities	Facilitators and barriers	Frequencyand duration
De Visschere et al. (2012) [31]	A supervised implementation of an oral healthcare guideline to improve the oral hygiene level of nursing home residents. *Organizational*: Conduction of one oral healthcare team per ward consisting of two oral healthcare organizers, a physician and either an occupational or speech therapist. *Professional:* 1.5 h presentation of the guideline, the oral healthcare protocol and the study to the director of nursing. 2 h theoretical and 1 h practical education for the members of the oral healthcare team covering the guideline.1.5 h training session for all ward nurses and nurse aids. Regularly bedside support of the oral healthcare organizers to ensure the delivery of the oral healthcare protocol and adherence to the guideline recommendations. Free oral healthcare products for all residents. Six-weekly meetings of the investigator, the project supervisor and the oral healthcare organizers to ensure implementation and to discuss problems.	Healthcare personnel, nursing home management.	Guideline dissemination^a^	*Multifaceted:* Clinical multidisciplinary teams, local consensus process, distribution of educational materials, education meetings, patient incentives.	Not prospectively identified.	Once in the beginning of the 6 months intervention period. Bedside-support and team meetings frequently over the 6 months intervention period.
Köpke et al. (2012) [32]	A multifaceted guideline implementation based on the theory of planned behaviour to reduce physical restraint use. *Professional:* 90 min. information session for intervention nursing homes to sensitize nurses about the matter of physical restraints and the message of the guideline by addressing their attitudes and experiences. Provision of a short version of the guideline. Distribution of posters, pens, mugs and notepads with the intervention’s logo. Flyers and brochures for relatives. Workshop for cluster-nurses on their role in the implementation process and in-depth information on avoiding physical restraints. A poster in the nursing homes foyer showing the contact nurses of the residents.	Healthcare personnel	Care as usual. Standard information provided: three brochures about the use of physical restraints and how to avoid them. A short presentation on physical restraints.	*Multifaceted:* Distribution of educational materials, education meetings, provision of promotional material.	Not prospectively identified.	Once in the beginning of the 6 months intervention period.
Tjia et al. (2015) [36]	A multifaceted, toolkit-based guideline implementation to reduce atypical antipsychotic prescribing rates. *Professional:* Arm 2: Mailed toolkit delivery with quarterly audit and feedback reports presenting aggregated facility-level data on atypical antipsychotic prescribing rates including benchmark comparisons with state and national prescribing levels. Provision of guideline-based information on efficacy and safety of atypical antipsychotics. Arm 3: On-site toolkit delivery with quarterly audit and feedback reports presenting aggregated facility-level data on atypical antipsychotic prescribing rates including benchmark comparisons with state and national prescribing levels. Provision of guideline-based information on efficacy and safety of atypical antipsychotics. Academic detailing for prescribers. One educational session for nurses and one for certified nurse assistants on the use of antipsychotics in nursing homes. Pharmacist meeting with the nursing home management to discuss important messages from the toolkit, ways to implement change and to get commitment statements on using the information and delivering it to the prescribers. Follow-up telephone call to discuss progress.	Healthcare personnel, nursing home management.	Arm 1: Mailed toolkit delivery (plain dissemination).	*Multifaceted:* Distribution of educational materials, education meetings, audit and feedback, academic detailing.	Not prospectively identified.	Quarterly delivery of audit & feedback reports, a single education meeting and multiple academic detailing visits during the 12 months intervention period. A single follow-up after 4–6 weeks after the academic detailing visits.
Van Gaal et al. (2011a/b) [33, 34]	Implementation of the patient safety programme “SAFE or SORRY?” to reduce the incidence of pressure ulcers, urinary tract infections and falls and to improve preventive care for residents at risk of those. *Professional:* 1.5 h small-scale education meetings on the wards for all nurses on the causes of pressure ulcers, urinary tract infections and falls, their prevention and on assessment of patients at risk. Two 30 min. case discussions on every ward on these topics. Distribution of a CD-ROM containing educational material and a knowledge test. Three separate information leaflets on the prevention of pressure ulcers, urinary tract infections and falls provided to residents at risk. Chart feedback on process and outcome indicators for the three adverse events using a computerized registration system.	Healthcare personnel.	Care as usual.	*Multifaceted:* Distribution of educational materials, education meetings, audit and feedback.	Not prospectively identified.	Once in the beginning of the 14 months intervention period. Chart feedback frequently over the 14 months intervention period.
Ward et al. (2010) [35]	Employment of a project nurse to encourage the adoption of best-practice falls prevention strategies. *Organizational:* Employment of a project nurse to encourage the facilities in using guideline-based strategies in fall risk and mobility assessment, the use of hip protectors, vitamin D supplementation, continence management, exercise programs, the use of appropriate footwear, medication review and post-fall management review. *Professional:* Provision of information on the prevention of falls and fall injuries to the intervention nursing homes. An initial training session followed by three-monthly network meetings. Development of a resource set to promote fall prevention guidelines. Workshop on running exercise programs for the healthcare personnel of the intervention facilities.	Healthcare personnel.	Care as usual.	*Multifaceted:* Clinical multidisciplinary teams, distribution of educational materials, education meetings.	Not prospectively identified.	Once in the beginning of the 17 months intervention period. Three-monthly network meetings over the 17 months intervention period.

^a^Not stated in the article. Information obtained via email from the corresponding author Luc De Visschere

### Risk of bias and overall quality

Using GRADE, the overall quality of the evidence for all outcomes was rated low or very low. We downgraded three studies due to a high risk of bias [33–36]. In addition, imprecision led to a downgrade in all included studies. According to GRADE, determinants for imprecise results are large confidence intervals, single studies, few events and small sample sizes [37]. Details on our risk of bias and GRADE assessment are available in the Additional files 4, 5, 6. Table 3 provides a short overview over the risk of bias in the included studies.

**Table 3:**
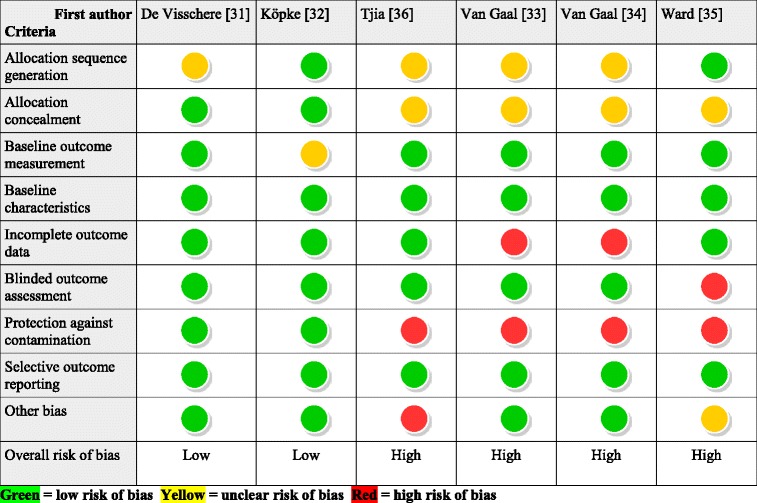
Risk of bias in included studies

### Effects on professional practice

Four studies evaluated the effects of guideline implementation strategies on professional practice [32, 34–36]. A total of 9453 residents with mean age of 83 years from 172 nursing homes participated in the studies.

Köpke and colleagues [32] examined the impact of a guideline implementation strategy based on the theory of planned behaviour on physical restraints use in nursing home residents. The intervention consisted of an information session, the provision of a short version of the guideline, a workshop, the distribution of promotional material and posters. Among the intervention sites, there was a statistically significant lower use of physical restraints (RR 0.78; 95 % CI: 0.63–0.97; *P =* 0.024) (Additional file 4: Table S1).

Tjia and colleagues [36] evaluated the effects of two different multifaceted, toolkit-based interventions on the prevalence of atypical antipsychotic use. The interventions consisted of audit and feedback, education meetings, educational material and academic detailing. In academic detailing, sometimes referred to as educational outreach, trained persons visit healthcare personnel in their workplaces and provide them with information on how they can improve their practice [17]. No difference in prescribing of atypical antipsychotics was reported for the intervention groups compared to the control group (Additional file 4: Tables S2, S3).

Van Gaal and colleagues [34] tested the effect of the patient safety programme “SAFE OR SORRY?” on the amount of adequate preventive care for residents at risk of pressure ulcers, urinary tract infections or falls. “SAFE OR SORRY?” consisted of a multifaceted guideline implementation strategy to implement three guidelines at once. The intervention included education meetings, distribution of educational material, case discussions and chart feedback. The risk ratio between the intervention and control group showed a non-significant increase in adequate care to prevent pressure ulcers (RR 1.60; 95 % CI: 0.94–2.76; *P =* 0.084) and urinary tract infections (RR 1.09; 95 % CI: 0.90–1.32; *P =* 0.37). There were too few events on the prevention of falls for a statistical analysis (Additional file 4: Table S4).

Ward and colleagues [35] evaluated the effect of employing a project nurse to facilitate the implementation of best-practice fall prevention on the use of vitamin D plus calcium supplements and hip protectors. The intervention is composed of an initial training session, network meetings, a resource set to promote fall prevention guidelines and a workshop. No differences were measured for both outcomes (Additional file 4: Table S5).

The overall quality of the studies for the results in the category *professional practice* was rated low [32] and very low [34–36] (Additional file 4: Tables S1-S5).

### Effects on patient outcome

Three studies evaluated the effects of guideline implementation strategies on patient outcome [31, 33, 35]. A total of 6080 residents with mean age of 82.5 years from 106 nursing homes participated in the studies.

De Visschere and colleagues [31] tested the effect of a supervised implementation of an oral healthcare guideline on the oral hygiene level of the participating residents. The intervention involved an oral healthcare team, guideline presentation, interactive education, training sessions, bedside support, network meetings and the provision of free oral healthcare products. Mean difference between the intervention and control group was a statistically non-significant reduction of tongue plaque (MD −0.07; 95 % CI: −0.91, 0.77; *P =* 0.87) and dental plaque (MD −0.15; 95 % CI: −0.45, 0.14; *P =* 0.31). The intervention nursing homes encountered a statistically significant reduction in denture plaque (MD −0.32; 95 % CI: −0.52, −0.11; *P =* 0.02) (Additional file 5: Table S6).

Van Gaal and colleagues [33] tested the effect of the patient safety programme “SAFE OR SORRY?” on the incidence of pressure ulcers, urinary tract infections and falls. The intervention evaluated in this study is described previously in the section “Effects on professional practice”. There was a statistically significant reduction of adverse events per patient week in favour of the intervention nursing homes (Rate ratio 0.67: 95 % CI: 0.47–0.97; *P <* 0.05) (Additional file 5: Table S7).

Ward and colleagues [35] evaluated the effect of employing a project nurse to facilitate the implementation of best-practice fall prevention on the rate of residents with at minimum one femoral neck fracture. The intervention evaluated in this study is described previously in the section “Effects on professional practice”. A statistically non-significant reduction in residents with at minimum one femoral neck fracture was measured in favour of the intervention group (RR 0.95; 95 % CI: 0.63–1.43; *P =* 0.79) (Additional file 5: Table S8).

The overall quality of the studies for the results in the category *patient outcome* was rated low [31] and very low [33, 35] (Additional file 4: Tables S6-S8).

## Discussion

This is the first systematic review to evaluate the effectiveness of guideline implementation strategies in nursing homes. This review includes five studies evaluating different multifaceted implementation strategies. No outcome was evaluated more than once, and different measures of effect were used. Thus, the results were not primarily comparable. The effects on professional practice and patient outcome were small to moderate and variable. The overall quality of the evidence was low or very low for each outcome, and our confidence in the results reported in these studies is therefore weak.

### Interventions to increase the implementation of guidelines in nursing homes

Köpke and colleagues [32] showed that theory-based guideline implementation can improve professional practice. However, despite the big sample size in this study, the effect estimate is imprecise and could vary from 38 % to nearly zero improvement. The imprecision can be explained by high intra-cluster correlation (ICC = 0.029) reducing the effective sample size. A multifaceted guideline implementation strategy to implement three guidelines at once, the employment of a project nurse to facilitate guideline implementation and a toolkit-based guideline implementation were not effective on professional practice [34–36]. The lack of effect may be due to contamination bias, which was present in all three studies. In the first study [34], participating nursing homes hosted wards from both the intervention- and control group. In the second [35] and third study [36], practice strategies targeting the primary study outcome were promoted nationwide during the intervention period, which may have influenced professional practice in the control group. In addition, general practitioners responsible for calcium and vitamin D prescription in the second study visited both intervention and control nursing homes [35].

De Visschere and colleagues [31] found a supervised guideline implementation to be effective on patient outcome. Yet, only one of three evaluated outcomes improved. The authors argue that sparse outcome-related events and the fact that the healthcare personnel disapproved one of the guideline recommendations could be responsible for the insufficient effectiveness. Van Gaal and colleagues [33] showed that a multifaceted guideline implementation strategy to implement three guidelines at once can improve patient outcome. However, the effect estimates are imprecise in both studies, with borderline significance on the lower end of the confidence interval caused by small sample sizes. As a consequence, although De Visschere and colleagues [31] and Van Gaal and colleagues [33] were able to document a statistically significant effect of their implementation strategies, the real effect may at worst be close to zero. The employment of a project nurse to facilitate guideline implementation was ineffective on patient outcome, most likely due to contamination bias as explained previously [35].

### Comparison with existing literature

Several EPOC reviews [14–17] evaluated guideline implementation strategies and have demonstrated that education meetings, printed educational materials, audit and feedback and academic detailing can improve professional practice and patient outcome. The overall effects were small and inconsistent with a median improvement of 16 % or less. Included studies in this review conform to the existing literature. Education meetings and printed educational materials were a part of the implementation strategies of all included studies, and audit and feedback was used in one study. But as the results were small to moderate and varied both within and across the included studies, it was impossible to determine which components were effective and to what degree.

The use and effectiveness of multifaceted implementation strategies is another often debated issue. All included studies used multifaceted implementation strategies. Their effects were small to moderate and variable, which concurs with evidence from multiple systematic reviews reporting on the topic [9, 11, 38]. Notably in this context is that the multifaceted implementation strategy in one of the included studies only improved patient outcome [33], but not professional practice [34], most probably due to contamination bias leading to a reduced measurable effect on professional practice. The causal relationship between guideline implementation and patient outcome is thus debatable. Many factors which are common in nursing homes can have impact on patient outcome as a measure for guideline implementation. The progress of slowly improving conditions as for example pressure ulcers or mobility after a hip fracture could take a long time before improvement may be measured, despite successful guideline implementation. Varying regularity and skills in applying guideline recommendations can also significantly reduce the effect of guideline recommendations on patient outcome [39, 40]. Professional practice directly depicts the extent of activities in concordance with recommendations from guidelines and may be better suited to measure guideline implementation in nursing homes, especially in studies where intervention follow-up is rather short.

### Limitations

The first and main limitation of this systematic review is the overall quality of the included evidence, which limits the strength of any conclusion. Second, only five studies were included, and every comparison was only evaluated once. We could therefore not identify any pattern that could have reliably linked the interventions to their outcomes. Third, clinical heterogeneity between the included studies prevented meta-analysis. We were therefore required to use a narrative approach, which is merely a coarse estimate of effect. Fourth, we applied a limiter excluding MEDLINE-indexed articles from the search results in some of the databases. We also excluded some possibly relevant articles because we were unable to determine the evidence base of the guidelines to be implemented. As a consequence, we may have missed relevant studies. Finally, we used the EPOC taxonomy of interventions to classify intervention components. Despite its widespread use, there is no general consensus on the use of this method to categorise intervention components in nursing homes.

### Implications for practice and future research

The impact on the field of practice of this systematic review is limited by sparse and low quality evidence. But this does not implicitly mean that the evaluated implementation strategies are ineffective. In fact, more high quality nursing home implementation studies are needed to establish a larger and more reliable evidence base. The multitude of quality improvement studies evaluating the impact of guidelines on patient outcome clearly shows the high interest in effective and reliable evidence in nursing homes. However, in order to improve patient outcome, guidelines must be implemented first. Thus, although patient outcomes are important and should be measured and reported, future studies evaluating interventions to improve guideline implementation should have a greater emphasis on outcomes that directly reflect change in guideline use.

There is also unused potential in the design of implementation strategies. Although highly recommended [10, 41, 42], none of the included studies identified and addressed barriers to change when tailoring their interventions. Moreover, not knowing the particular barriers to change precludes proper identification of the factors that rendered an implementation strategy ineffective. And finally, yet rarely used, the use of behavioural theory when designing an implementation strategy may be another promising approach to change behaviour towards guideline use [43, 44]. It may also be an explanation for the successful guideline implementation in one of the included studies despite its short study period, where the theory of planned behaviour was applied to the implementation strategy [32].

## Conclusions

There are few studies which can inform practice in nursing homes on how to successfully implement guidelines. We identified six different multifaceted interventions targeting six different outcomes. The effects of the guideline implementation strategies included in this review are small to moderate, are variable and concur with the body of evidence from other settings. The overall quality of the evidence was low or very low for all comparisons in this review. On that basis, it is not possible to recommend or discourage the use of a particular guideline implementation strategy. Rather, these findings illustrate a large evidence gap. More implementation research is needed to ensure high quality of care in nursing homes.

Care providers in nursing homes and researchers should carefully identify and address barriers to change when designing their implementation strategies. Authors of future studies are encouraged to focus on outcomes that directly reflect guideline implementation. The use of behavioural theory when designing an implementation strategy should be studied further.

## Abbreviations

EPOC, the cochrane effective practice and organization of care group, GRADE, the grading of recommendations, assessment, development and evaluations, KT, knowledge translation.

## Additional files

Additional file 1: Search strategy. The complete search strategies for all searched databases. (DOCX 35 kb) Additional file 2: Data abstraction form. The customised EPOC data abstraction form. (PDF 93 kb) Additional file 3: Table of excluded studies. A list of excluded studies from which the reader might have expected to find in this review together with a rationale for exclusion. (PDF 105 kb) Additional file 4: Summary of findings tables (professional practice). Summary of findings tables for outcomes classified as professional practice. (PDF 84 kb) Additional file 5: Summary of findings tables (patient outcome). Summary of findings tables for outcomes classified as patient outcome. (PDF 77 kb) Additional file 6: Risk of bias assessment. A detailed description of the risk of bias assessment of all included studies. (PDF 138 kb)
