# Supplementary material for: Implementing guidelines in nursing homes: a systematic review
Source: BMC Health Serv Res. 2016 Jul 25;16:298. doi: 10.1186/s12913-016-1550-z (PMC4960750; doi:10.1186/s12913-016-1550-z)
Supplement: Additional file 1: — Search strategy. The complete search strategies for all searched databases. (DOCX 35 kb) [file 12913_2016_1550_MOESM1_ESM.docx]

**Additional file 1 – Search strategy**

| Search strategy: CINAHL (EBSCOhost)  Timespan: 1984 – August 2015  Search date: 23.08.2015  Hits: 234 |
| --- |

| S1 | (MH "Nursing Homes+") |
| --- | --- |
| S2 | TI (nursing W0 (home# or facilit*")) or AB (nursing W0 (home# or facilit*")) |
| S3 | TI (intermediate or long-term or longterm) W0 ("care facilit*") or AB (intermediate or long-term or longterm) W0 ("care facilit*") |
| S4 | TI (("aged care" or "skilled nursing") W0 facilit*) or AB (("aged care" or "skilled nursing") W0 facilit*) |
| S5 | TI ("home# for the aged" or "home# for the elderly") or AB ("home# for the aged" or "home# for the elderly") |
| S6 | S1 OR S2 OR S3 OR S4 OR S5 |
| S7 | (MH "Practice Guidelines") |
| S8 | (MH "Guideline Adherence") |
| S9 | (MH "Professional Compliance") |
| S10 | TI ((guideline# or protocol) N2 (implementation or dissemination or uptake or diffusion or adherence or compliance)) or AB ((guideline# or protocol) N2 (implementation or dissemination or uptake or diffusion or adherence or compliance)) |
| S11 | (MH "Professional Practice, Evidence-Based+") |
| S12 | TI ("evidence based") W0 (practice or nursing or medicine) or AB ("evidence based") W0 (practice or nursing or medicine) |
| S13 | TI (evidence N2 uptake) or AB (evidence N2 uptake) |
| S14 | (MH "Selective Dissemination of Information") |
| S15 | TI (information or "best practice" or guideline# or research) N2 (dissemination or utili?ation) or AB (information or "best practice" or guideline# or research) N2 (dissemination or utili?ation) |
| S16 | TI "effective dissemination#" or AB "effective dissemination#" |
| S17 | TI (applied W0 (dissemination or "health research")) or AB (applied W0 (dissemination or "health research")) |
| S18 | (MH "Diffusion of Innovation") |
| S19 | TI (innovation N2 (adaptation or adoption or diffusion)) or AB (innovation N2 (adaptation or adoption or diffusion)) |
| S20 | TI "best practice#" or AB "best practice#" |
| S21 | TI "capacity building" or AB "capacity building" |
| S22 | TI (change N2 implementation#) or AB (change N2 implementation#) |
| S23 | TI ((changing W0 (provider or physician or doctor)) W0 behavio#r) or AB ((changing W0 (provider or physician or doctor)) W0 behavio#r) |
| S24 | TI "collaborative development" or AB "collaborative development" |
| S25 | TI (complex W0 (intervention# or science# or study or studies)) or AB (complex W0 (intervention# or science# or study or studies)) |
| S26 | TI ((continuing W0 (medical or nursing or dental)) W0 education#) or AB ((continuing W0 (medical or nursing or dental)) W0 education#) |
| S27 | TI "crossing the quality chasm" or AB "crossing the quality chasm" |
| S28 | TI ((effectiveness or evaluation) W0 research*) or AB ((effectiveness or evaluation) W0 research*) |
| S29 | TI (gap N2 (analysis or evidence or practice)) or AB (gap N2 (analysis or evidence or practice)) |
| S30 | TI (audit N2 feedback) or AB (audit N2 feedback) |
| S31 | TI ((getting W0 (knowledge or research)) W0 "into practice") or AB ((getting W0 (knowledge or research)) W0 "into practice") |
| S32 | TI "GRIP" or AB "GRIP"^[[1]](#footnote-1)^ |
| S33 | TI "know-do" or AB "know-do" |
| S34 | TI (knowledge N2 (adoption or brokering or communication or cycle# or developement or application or diffusion or dissemination or exchange or management or mobili?ation or synthesis or transfer or transformation or translation or uptake or utili?ation)) or AB (knowledge N2 (adoption or brokering or communication or cycle# or developement or application or diffusion or dissemination or exchange or management or mobili?ation or synthesis or transfer or transformation or translation or uptake or utili?ation)) |
| S35 | TI "knowledge to action" or AB "knowledge to action" |
| S36 | TI "KSTE" or AB "KSTE"^[[2]](#footnote-2)^ |
| S37 | TI ("linkage and exchange") or AB ("linkage and exchange") |
| S38 | TI "opinion leader#" or AB "opinion leader#" |
| S39 | TI (patient W0 (education or safety)) or AB (patient W0 (education or safety)) |
| S40 | TI "populari?ation of research" or AB "populari?ation of research" |
| S41 | TI "professional behavio#r change" or AB "professional behavio#r change" |
| S42 | TI (quality W0 (assurance or improv*)) or AB (quality W0 (assurance or improv*)) |
| S43 | TI (research N2 (capacity or implementation or mediation or transfer or translation or utili?ation)) or AB (research N2 (capacity or implementation or mediation or transfer or translation or utili?ation)) |
| S44 | TI ("research into" W0 (action or practice)) or AB ("research into" W0 (action or practice)) |
| S45 | TI "science communication" or AB "science communication" |
| S46 | TI (quality N2 improvement) or AB (quality N2 improvement) |
| S47 | TI ((technology or technologies) N2 transfer) or AB ((technology or technologies) N2 transfer) |
| S48 | TI ((translat* or turning) W0 research) or AB ((translat* or turning) W0 research) |
| S49 | TI "TRIP" or AB "TRIP"^[[3]](#footnote-3)^ |
| S50 | TI "translational science" or AB "translational science" |
| S51 | TI (third W0 (mission or wave)) or AB (third W0 (mission or wave)) |
| S52 | S7 OR S8 OR S9 OR S10 OR S11 OR S12 OR S13 OR S14 OR S15 OR S16 OR S17 OR S18 OR S19 OR S20 OR S21 OR S22 OR S23 OR S24 OR S25 OR S26 OR S27 OR S28 OR S29 OR S30 OR S31 OR S32 OR S33 OR S34 OR S35 OR S36 OR S37 OR S38 OR S39 OR S40 OR S41 OR S42 OR S43 OR S44 OR S45 OR S46 OR S47 OR S48 OR S49 OR S50 OR S51 |
| S53 | S6 AND S52 |
| S54 | PT clinical trial |
| S55 | PT research |
| S56 | (MH "Randomized Controlled Trials") |
| S57 | (MH "Clinical Trials") |
| S58 | (MH "Intervention Trials") |
| S59 | (MH "Nonrandomized Trials") |
| S60 | (MH "Experimental Studies") |
| S61 | (MH "Pretest-Posttest Design+") |
| S62 | (MH "Quasi-Experimental Studies+") |
| S63 | (MH "Multicenter Studies") |
| S64 | (MH "Health Services Research") |
| S65 | TI ( randomis* or randomiz* or random* W0 allocat* ) OR AB ( randomis* or randomiz* or random* W0 allocat* ) |
| S66 | TI ( (intervention* or controlled or control W0 group* or compare or compared or before N5 after or pre N5 post or pretest or "pre test" or posttest or "post test" or quasiexperiment* or quasi W0 experiment* or evaluat* or effect or impact or "time series" or time W0 point* or repeated W0 measur*) ) OR AB ( (intervention* or controlled or control W0 group* or compare or compared or before N5 after or pre N5 post or pretest or "pre test" or posttest or "post test" or quasiexperiment* or quasi W0 experiment* or evaluat* or effect or impact or "time series" or time W0 point* or repeated W0 measur*) ) |
| S67 | TX meta-analysis |
| S68 | PT review |
| S69 | PT systematic review |
| S70 | S54 OR S55 OR S56 OR S57 OR S58 OR S59 OR S60 OR S61 OR S62 OR S63 OR S64 OR S65 OR S66 OR S67 OR S68 OR S69 |
| S71 | S53 AND S70 Limiters - Exclude MEDLINE records |

| Search strategy: ClinicalTrials  Timespan: All years  Search date: 23.08.2015  Hits: 37 |
| --- |

(nursing home OR intermediate care facility OR long term care facility OR skilled nursing facility OR home for the aged) AND (guideline (implementation OR dissemination OR uptake OR diffusion OR adherence OR translation))

| Search strategy: Cochrane Database of Systematic Reviews (CDSR), Cochrane Central Register of Controlled Trials (CENTRAL)  Timespan: All years  Search date: 23.08.2015  Hits: 223 |
| --- |

#1 MeSH descriptor: [Nursing Homes] explode all trees

#2 (nursing next (home? or facilit*)):ti,ab,kw

#3 ((intermediate or long-term or longterm) next "care facilit*"):ti,ab,kw

#4 ((aged-care or "aged care" or skilled-nursing or "skilled nursing") next facilit*):ti,ab,kw

#5 MeSH descriptor: [Homes for the Aged] explode all trees

#6 ("home? for the aged" or "home? for the elderly"):ti,ab,kw

#7 #1 or #2 or #3 or #4 or #5 or #6

#8 MeSH descriptor: [Guideline] explode all trees

#9 MeSH descriptor: [Guidelines as Topic] explode all trees

#10 MeSH descriptor: [Guideline Adherence] explode all trees

#11 (guideline near/2 (implementation or dissemination or uptake or diffusion or adherence)):ti,ab,kw

#12 MeSH descriptor: [Evidence-Based Practice] explode all trees

#13 MeSH descriptor: [Evidence-Based Nursing] explode all trees

#14 MeSH descriptor: [Evidence-Based Medicine] explode all trees

#15 (("evidence based" or "evidence based") next (nursing or medicine or practice)):ti,ab,kw

#16 (evidence near/2 uptake):ti,ab,kw

#17 MeSH descriptor: [Information Dissemination] explode all trees

#18 ((information or "best practice" or guideline? or research) near/2 (dissemination or utili?ation)):ti,ab,kw

#19 "effective dissemination":ti,ab,kw

#20 (applied next (dissemination or "health research")):ti,ab,kw

#21 MeSH descriptor: [Diffusion of Innovation] explode all trees

#22 (innovation near/2 (adaptation or adoption or diffusion)):ti,ab,kw

#23 "best practice?":ti,ab,kw

#24 "capacity building":ti,ab,kw

#25 (change near/2 implementation?):ti,ab,kw

#26 (changing next ((provider or physician or doctor) next behavio?r))

#27 "collaborative development":ti,ab,kw

#28 (complex next (intervention? or science? or study or studies)):ti,ab,kw

#29 (continuing next ((medical or nursing or dental) next education*)):ti,ab,kw

#30 "crossing the quality chasm":ti,ab,kw

#31 ((effectiveness or evaluation) next research*):ti,ab,kw

#32 (gap near/2 (analysis or evidence or practice)):ti,ab,kw

#33 (audit near/2 feedback):ti,ab,kw

#34 (getting next (knowledge or research) next "into practice"):ti,ab,kw

#35 GRIP:ti,ab,kw

#36 know-do:ti,ab,kw

#37 (Knowledge near/2 (adoption or brokering or communication or cycle? or developement or application or diffusion or dissemination or exchange or management or mobili?ation or synthesis or transfer or transformation or translation or uptake or utili?ation)):ti,ab,kw

#38 "knowledge to action":ti,ab,kw

#39 KSTE:ti,ab,kw

#40 "linkage and exchange":ti,ab,kw

#41 "opinion leader?":ti,ab,kw

#42 (patient next (education or safety)):ti,ab,kw

#43 "populari?ation of research":ti,ab,kw

#44 "professional behavio?r change":ti,ab,kw

#45 (quality near/2 (assurance or improv*)):ti,ab,kw

#46 (research near/2 (capacity or implementation or mediation or transfer or translation or utili?ation)):ti,ab,kw

#47 "research into (action or practice)":ti,ab,kw

#48 "science communication":ti,ab,kw

#49 (quality near/2 improvement?):ti,ab,kw

#50 ((technology or technologies) near/2 transfer):ti,ab,kw

#51 ((translat* or turning) next research):ti,ab,kw

#52 TRIP:ti,ab,kw

#53 "translational science":ti,ab,kw

#54 (third next (mission or wave)):ti,ab,kw

#55 #8 or #9 or #10 or #11 or #12 or #13 or #14 or #15 or #16 or #17 or #18 or #19 or #20 or #21 or #22 or #23 or #24 or #25 or #26 or #27 or #28 or #29 or #30 or #31 or #32 or #33 or #34 or #35 or #36 or #37 or #38 or #39 or #40 or #41 or #42 or #43 or #44 or #45 or #46 or #47 or #48 or #49 or #50 or #51 or #52 or #53 or #54

#56 #7 and #55

| Search strategy: Database of Abstracts of Reviews of Effects (DARE), Health Assessment Database (HTA)  Timespan: All years  Search date: 23.08.2015  Hits: 19 |
| --- |

1. MeSH DESCRIPTOR Nursing Homes EXPLODE ALL TREES

2. (“intermediate care” OR “long term care”): TI IN DARE, HTA

3. (“aged care facilit*” OR “skilled nursing facilit*”): TI IN DARE, HTA

4. MeSH DESCRIPTOR Homes for the Aged EXPLODE ALL TREES

5. (“home* for the aged” OR “home* for the elderly”): TI IN DARE, HTA

6. #1 OR #2 OR #3 OR #4 OR #5

7. MeSH DESCRIPTOR Guideline EXPLODE ALL TREES

8. MeSH DESCRIPTOR Guidelines as Topic EXPLODE ALL TREES

9. MeSH DESCRIPTOR Guideline Adherence EXPLODE ALL TREES

10. (implementation OR dissemination OR uptake OR diffusion OR adherence OR translation): TI IN DARE, HTA

11. MeSH DESCRIPTOR Evidence-Based Practice EXPLODE ALL TREES

12. MeSH DESCRIPTOR Evidence-Based Nursing EXPLODE ALL TREES

13. MeSH DESCRIPTOR Evidence-Based Medicine EXPLODE ALL TREES

14. (“evidence based”): TI IN DARE, HTA

15. MeSH DESCRIPTOR Information Dissemination EXPLODE ALL TREES

16. MeSH DESCRIPTOR Diffusion of Innovation EXPLODE ALL TREES

17. #7 OR #8 OR #9 OR #10 OR #11 OR #12 OR #13 OR #14 OR #15 OR #16

18. #6 AND #17

| Search strategy: Embase (Ovid)  Timespan: 1974 – August 2015  Search date: 23.08.2015  Hits: 376 |
| --- |

1. exp nursing home/

2. (nursing adj (home? or facilit*)).tw.

3. ((intermediate or long-term or longterm) adj care facilit*).tw.

4. ((aged-care or skilled-nursing) adj facilit*).tw.

5. exp home for the aged/

6. (home? for the aged or home? for the elderly).tw.

7. 1 or 2 or 3 or 4 or 5 or 6

8. exp practice guideline/

9. exp protocol compliance/

10. ((guideline? or protocol) adj2 (implementation or dissemination or uptake or diffusion or adherence or compliance)).tw.

11. exp evidence based practice/

12. exp evidence based medicine/

13. (evidence-based adj (nursing or medicine or practice)).tw.

14. (evidence adj2 uptake).tw.

15. exp information dissemination/

16. ((information or "best practice" or guideline? or research) adj2 (dissemination or utili? ation)).tw.

17. "effective dissemination?".tw.

18. (applied adj (dissemination or health research)).tw.

19. (innovation adj2 (adaptation or adoption or diffusion)).tw.

20. "best practice?".tw.

21. "capacity building".tw.

22. (change adj2 implementation?).tw.

23. (changing adj ((provider or physician or doctor) adj behavio?r)).tw.

24. "collaborative development".tw.

25. (complex adj (intervention? or science? or study or studies)).tw.

26. (continuing adj ((medical or nursing or dental) adj education*)).tw.

27. "crossing the quality chasm".tw.

28. ((effectiveness or evaluation) adj research*).tw.

29. (gap adj2 (analysis or evidence or practice)).tw.

30. (audit adj2 feedback).tw.

31. (getting adj (knowledge or research) adj into practice).tw.

32. GRIP.tw.

33. know-do.tw.

34. (Knowledge adj2 (adoption or brokering or communication or cycle? or developement or application or diffusion or dissemination or exchange or management or mobili?ation or synthesis or transfer or transformation or translation or uptake or utili?ation)).tw.

35. "knowledge to action".tw.

36. KSTE.tw.

37. "linkage and exchange".tw.

38. "opinion leader?".tw.

39. (patient adj (education or safety)).tw.

40. "populari?ation of research".tw.

41. "professional behavio?r change".tw.

42. (quality adj2 (assurance or improv*)).tw.

43. (research adj2 (capacity or implementation or mediation or transfer or translation or utili? ation)).tw.

44. "research into (action or practice)".tw.

45. "science communication".tw.

46. (quality adj2 improvement?).tw.

47. ((technology or technologies) adj2 transfer).tw.

48. ((translat* or turning) adj research).tw.

49. TRIP.tw.

50. "translational science".tw.

51. (third adj (mission or wave)).tw.

52. 8 or 9 or 10 or 11 or 12 or 13 or 14 or 15 or 16 or 17 or 18 or 19 or 20 or 21 or 22 or 23 or 24 or 25 or 26 or 27 or 28 or 29 or 30 or 31 or 32 or 33 or 34 or 35 or 36 or 37 or 38 or 39 or 40 or 41 or 42 or 43 or 44 or 45 or 46 or 47 or 48 or 49 or 50 or 51

53. 7 and 52

54. Randomized Controlled Trial/

55. Controlled Clinical Trial/

56. Quasi Experimental Study/

57. Pretest Posttest Control Group Design/

58. Time Series Analysis/

59. Experimental Design/

60. Multicenter Study/

61. (randomis* or randomiz* or randomly or random allocat*).ti,ab.

62. groups.ab.

63. (trial or multicentre or multicenter or multi centre or multi center).ti.

64. (intervention* or controlled or control group or compare or compared or (before adj5 after) or (pre adj5 post) or pretest or pre test or posttest or post test or quasiexperiment* or quasi experiment* or evaluat* or effect or impact or time series or time point? or repeated measur*).ti,ab.

65. or/54-64

66. Nonhuman/

67. 65 not 66

68. meta-analy:.mp.

69. search:.tw.

70. review.pt.

71. or/68-70

72. 67 or 71

73. 53 and 72

74. limit 73 to exclude medline journals

| Search strategy: ISI Web of Science  Timespan: All years  Search date: 23.08.2015  Hits: 390 |
| --- |

#1 **TOPIC:** ("nursing home" or "nursing homes" or "nursing facilit*")

#2 **TOPIC:** ("intermediate care facilit*" or "long-term care facilit*" or "longterm care facilit*")

#3 **TOPIC:** ("aged-care facilit*" or "skilled-nursing facilit*")

#4 **TOPIC:** ("home for the aged" or "homes for the aged")

#5 **TOPIC:** ("home for the elderly" or "homes for the elderly")

#6 #1 OR #2 OR #3 OR #4 OR #5

#7 **TOPIC:** (guideline$)

#8 **TOPIC:** ("evidence-based practice" or "evidence-based nursing" or "evidence-based medicine")

#9 **TOPIC:** (evidence NEAR/2 uptake)

#10 **TOPIC:** ((information or "best practice" or guideline$ or research) NEAR/2 (dissemination or utili?ation))

#11 **TOPIC:** ("effective dissemination")

#12 **TOPIC:** ("applied dissemination" or "applied health research")

#13 **TOPIC:** (innovation NEAR/2 (adaptation or adoption or diffusion))

#14 **TOPIC:** ("best practice" or "best practices")

#15 **TOPIC:** ("capacity building")

#16 **TOPIC:** (change NEAR/2 implementation)

#17 **TOPIC:** ("changing provider behavior" or "changing physician behavior" or "changing doctor behavior")

#18 **TOPIC:** ("changing provider behaviour" or "changing physician behaviour" or "changing doctor behaviour")

#19 **TOPIC:** ("collaborative development")

#20 **TOPIC:** ("complex intervention" or "complex interventions" or "complex science" or "complex sciences" or "complex study" or "complex studies")

#21 **TOPIC:** ("continuing medical education" or "continuing nursing education" or "continuing dental education")

#22 **TOPIC:** ("crossing the quality chasm")

#23 **TOPIC:** ("effectiveness research" or "evaluation research")

#24 **TOPIC:** (gap NEAR/2 (analysis or evidence or practice))

#25 **TOPIC:** (audit NEAR/2 feedback)

#26 **TOPIC:** ("getting knowledge into practice" or "getting research into practice")

#27 **TOPIC:** (GRIP)

#28 **TOPIC:** ("know-do")

#29 **TOPIC:** (knowledge NEAR/2 (adoption or brokering or communication or cycle$ or developement or application or diffusion or dissemination or exchange or management or mobili?ation or synthesis or transfer or transformation or translation or uptake or utili?ation))

#30 **TOPIC:** ("knowledge to action")

#31 **TOPIC:** (KSTE)

#32 **TOPIC:** ("linkage and exchange")

#33 **TOPIC:** ("opinion leader" or "opinion leaders")

#34 **TOPIC:** ("patient education" or "patient safety")

#35 **TOPIC:** ("populari?ation of research")

#36 **TOPIC:** ("professional behavior change" or "professional behaviour change")

#37 **TOPIC:** (quality NEAR/2 (assurance or improv*))

#38 **TOPIC:** (research NEAR/2 (capacity or implementation or mediation or transfer or translation or utili?ation))

#39 **TOPIC:** ("research into action" or "research into practice")

#40 **TOPIC:** ("science communication")

#41 **TOPIC:** (quality NEAR/2 improvement)

#42 **TOPIC:** ((technology or technologies) NEAR/2 transfer)

#43 **TOPIC:** ("translat* research" or "turning research")

#44 **TOPIC:** (TRIP)

#45 **TOPIC:** ("translational science")

#46 **TOPIC:** ("third mission" or "third wave")

#47 #7 OR #8 OR #9 OR #10 OR #11 OR #12 OR #13 OR #14 OR #15 OR #16 OR #17 OR #18 OR #19 OR #20 OR #21 OR #22 OR #23 OR #24 OR #25 OR #26 OR #27 OR #28 OR #29 OR #30 OR #31 OR #32 OR #33 OR #34 OR #35 OR #36 OR #37 OR #38 OR #39 OR #40 OR #41 OR #42 OR #43 OR #44 OR #45 OR #46

#48 #6 AND #47

#49 **TOPIC:** ((random* or “control* trial*” or intervention* or experiment* or “time series” or “pre test” or pretest or “post test” or posttest or impact* or chang* or evaluat* or effect* or comparat*))

#50 **TOPIC:** (review)

#51 **TOPIC:** ("meta-analysis")

#52 **TOPIC:** (search)

#53 #50 OR #51 OR #52

#54 #49 OR #53

#55 #48 AND #54 Refined by: [excluding] Databases=( MEDLINE )

| Search strategy: MEDLINE ® In-Process & Other Non-Indexed Citations and Ovid MEDLINE ®  Timespan: 1946 – August 2015  Search date: 23.08.2015  Hits: 2017 |
| --- |

1. exp Nursing Homes/

2. (nursing adj (home? or facilit*)).tw.

3. ((intermediate or long-term or longterm) adj care facilit*).tw.

4. ((aged-care or skilled-nursing) adj facilit*).tw.

5. exp Homes for the Aged/

6. (home? for the aged or home? for the elderly).tw.

7. or/1-6

8. exp Guideline/

9. exp Guidelines as Topic/

10. exp Guideline Adherence/

11. ((guideline? or protocol) adj2 (implementation or dissemination or uptake or diffusion or adherence or compliance)).tw.

12. exp Evidence-Based Practice/

13. exp Evidence-Based Nursing/

14. exp Evidence-based Medicine/

15. (evidence-based adj (nursing or medicine or practice)).tw.

16. (evidence adj2 uptake).tw.

17. exp Information Dissemination/

18. ((information or "best practice" or guideline? or research) adj2 (dissemination or utili? ation)).tw.

19. "effective dissemination?".tw.

20. (applied adj (dissemination or health research)).tw.

21. exp Diffusion of Innovation/

22. (innovation adj2 (adaptation or adoption or diffusion)).tw.

23. "best practice?".tw.

24. "capacity building".tw.

25. (change adj2 implementation?).tw.

26. (changing adj ((provider or physician or doctor) adj behavio?r)).tw.

27. "collaborative development".tw.

28. (complex adj (intervention? or science? or study or studies)).tw.

29. (continuing adj ((medical or nursing or dental) adj education*)).tw.

30. "crossing the quality chasm".tw.

31. ((effectiveness or evaluation) adj research*).tw.

32. (gap adj2 (analysis or evidence or practice)).tw.

33. (audit adj2 feedback).tw.

34. (getting adj (knowledge or research) adj into practice).tw.

35. GRIP.tw.

36. know-do.tw.

37. (Knowledge adj2 (adoption or brokering or communication or cycle? or developement or application or diffusion or dissemination or exchange or management or mobili?ation or synthesis or transfer or transformation or translation or uptake or utili?ation)).tw.

38. "knowledge to action".tw.

39. KSTE.tw.

40. "linkage and exchange".tw.

41. "opinion leader?".tw.

42. (patient adj (education or safety)).tw.

43. "populari?ation of research".tw.

44. "professional behavio?r change".tw.

45. (quality adj2 (assurance or improv*)).tw.

46. (research adj2 (capacity or implementation or mediation or transfer or translation or utili? ation)).tw.

47. "research into (action or practice)".tw.

48. "science communication".tw.

49. (quality adj2 improvement?).tw.

50. ((technology or technologies) adj2 transfer).tw.

51. ((translat* or turning) adj research).tw.

52. TRIP.tw.

53. "translational science".tw.

54. (third adj (mission or wave)).tw.

55. or/8-54

56. 7 and 55

57. randomized controlled trial.pt.

58. controlled clinical trial.pt.

59. multicenter study.pt.

60. (randomis* or randomiz* or randomly allocat* or random allocat*).ti,ab.

61. groups.ab.

62. (trial or multicenter or multi center or multicentre or multi centre).ti.

63. (intervention* or controlled or control group or compare or compared or (before adj5 after) or (pre adj5 post) or pretest or pre test or posttest or post test or quasiexperiment* or quasi experiment* or evaluat* or effect or impact or time series or time point? or repeated measur*).ti,ab.

64. or/57-63

65. exp Animals/

66. Humans/

67. 65 not (65 and 66)

68. 64 not 67

69. review.ab.

70. review.pt.

71. meta-analysis.ab.

72. meta-analysis.pt.

73. meta-analysis.ti.

74. or/69-73

75. letter.pt.

76. comment.pt.

77. editorial.pt.

78. or/75-77

79. 74 not 78

80. 68 or 79

81. 56 and 80

| Search strategy: OpenGrey  Timespan: All years  Search date: 23.08.2015  Hits: 5 |
| --- |

(nursing home OR intermediate care facility OR long term care facility OR skilled nursing facility OR home for the aged) AND (implementation OR dissemination OR uptake OR diffusion OR adherence OR translation)

| Search strategy: PROSPERO  Timespan: All years  Search date: 23.08.2015  Hits: 64 |
| --- |

nursing home OR intermediate care facility OR long term care facility OR skilled nursing facility OR home for the aged

| Search strategy: SveMed+  Timespan: All years  Search date: 23.08.2015  Hits: 46 |
| --- |

1 exp:"nursing homes"

2 "nursing home" OR "nursing facility"

3 "intermediate care facility" OR "long term care facility

4 "aged care facility" OR "skilled nursing facility”

5 exp:"homes for the aged"

6 "home for the aged" OR "home for the elderly"

7 #1 OR #2 OR #3 OR #4 OR #5 OR #6

8 exp:"Guidelines as Topic"

9 exp:"Guideline Adherence”

10 implementation OR dissemination OR uptake OR diffusion OR adherence OR translation

11 exp:"Evidence-Based Practice"

12 exp:"Evidence-Based Nursing"

13 exp:"Evidence-Based Medicine”

14 "evidence based"

15 exp:"Information Dissemination"

16 exp:"Diffusion of Innovation"

17 #8 OR #9 OR #10 OR #11 OR #12 OR #13 OR #14 OR #15 OR #16

18 #7 AND #17

1. GRIP: Getting research into practice [↑](#footnote-ref-1)
2. KSTE: Knowledge translation, synthesis and exchange [↑](#footnote-ref-2)
3. TRIP: Translating research into practice [↑](#footnote-ref-3)
