# Supplementary material for: Implementing guidelines in nursing homes: a systematic review
Source: BMC Health Serv Res. 2016 Jul 25;16:298. doi: 10.1186/s12913-016-1550-z (PMC4960750; doi:10.1186/s12913-016-1550-z)
Supplement: Additional file 2: — Data abstraction form. The customised EPOC data abstraction form. (PDF 93 kb) [file 12913_2016_1550_MOESM2_ESM.pdf]

Additional file 2 – Data abstraction form

DATA ABSTRACTION FORM

Data collection

Name of reviewer:
Date:
Study ID:
Study reference:

Objective(s):

Scope:
The effect(s) of a professional, organisational, financial or regulatory intervention(s) to implement guidelines in nursing homes is evaluated. ☐

1. INCLUSION CRITERIA

1.1 Study design

**Cluster-randomised controlled trial**	☐
**Randomised controlled trial**	☐
**Controlled before-after design**	☐
**Interrupted-time-series design**	☐
Clearly defined entry point in time when the intervention occurred	☐
At least three data points before and three after the intervention	☐

1.2 Participants

**Healthcare personnel working in a nursing home**	☐

1.3 Intervention(s)

**Professional** ☐	**Organisational** ☐	**Financial** ☐	**Regulatory** ☐
**Intervention clearly stated** ☐			
**Based upon implementation of clinical practice guideline(s)** ☐			
**Clinical practice guideline(s)**<br>Based on a review of the literature ☐<br>Recommendations tied to the identified evidence ☐<br>Publicly available ☐			

1.4 Control intervention(s)

**Professional** ☐	**Organisational** ☐	**Financial** ☐	**Regulatory** ☐
**Care as usual** ☐			
**Other (specify):**			

1.5 Outcome(s)

**The objective measurement of performance / provider behaviour or health / patient outcomes** ☐
**Relevant and interpretable data presented or obtainable** ☐

2. INTERVENTIONS

2.1 Type of intervention (state all interventions for each comparison / study group)

2.2 Control(s)

**3. TYPE OF TARGETED BEHAVIOUR** (state more than one where appropriate)

**4. PARTICIPANTS**

4.1 Characteristics of participating providers

**Profession:**
**Level of vocational training:**

4.2 Characteristics of participating residents

4.2.1 Other resident characteristics

**Age:**
**Gender:**
**Ethnicity:**
**Other (specify):**

4.2.2 Number of residents included in the study

**Episodes of care:**
**Residents:**
**Providers:**
**Communities or regions:**

**5. SETTING**

**Location of care:**
**Country:**
**Proportion of eligible providers or allocation units:**

6. METHODS

**Unit of allocation:**
**Unit of analysis:**
**Power calculation:**

7. PROSPECTIVE IDENTIFICATION OF BARRIERS TO CHANGE

8. INTERVENTION

8.1 Characteristics of the intervention

**Evidence base of recommendation:**	
**Purpose of recommendations:**	
**Single intervention** ☐	**Multifaceted intervention** ☐

8.2 Timing

**Frequency / number of intervention events:**
**Duration of intervention:**

9. OUTCOMES

9.1 Description of the main outcome measure(s)

**Health professional outcomes / process measures:**
**Patient outcomes:**

9.2 Length of time

**Length of time during which outcomes were measured after initiation of the intervention:**
**Length of post-intervention follow-up:**

9.3 Identify a possible ceiling effect

**Identified by investigator**	☐
**Identified by reviewer**	☐

**10. RESULTS** (use extra page if necessary)

10.1.1 For (cluster-)randomised controlled trials

10.1.2 For controlled before-after studies

10.1.3 For interrupted-time-series studies

