# Supplementary material for: Implementing guidelines in nursing homes: a systematic review
Source: BMC Health Serv Res. 2016 Jul 25;16:298. doi: 10.1186/s12913-016-1550-z (PMC4960750; doi:10.1186/s12913-016-1550-z)
Supplement: Additional file 3: — Table of excluded studies. A list of excluded studies from which the reader might have expected to find in this review together with a rationale for exclusion. (PDF 105 kb) [file 12913_2016_1550_MOESM3_ESM.pdf]

Additional file 3 - Table of excluded studies

Study reference	Reason for exclusion
Beeckman D, Clays E, Van Hecke A, Vanderwee K, Schoonhoven L, Verhaeghe S: **A multi-faceted tailored strategy to implement an electronic clinical decision support system for pressure ulcer prevention in nursing homes: a two-armed randomized controlled trial.** *Int J Nurs Stud* 2013, **50**:475–86.	Self-developed computerised decision support system (PrevPlan), guidelines used not stated.
Chami K, Gavazzi G, Bar-Hen A, Carrat F, de Wazieres B, Lejeune B, Armand N, Rainfray M, Hajjar J, Piette F, Tondeur MR: **A short-term, multicomponent infection control program in nursing homes: a cluster randomized controlled trial.** *J Am Med Dir Assoc* 2012, **13**:569.e9–17.	Guideline based on a Delphi consensus survey. No review of the literature.
Colón-Emeric CS, Lyles KW, House P, Levine DA, Schenck AP, Allison J, Gorospe J, Fermazin M, Oliver K, Curtis JR, others: **Randomized trial to improve fracture prevention in nursing home residents**. *Am J Med* 2007, **120**:886–892.	Excluded after risk of bias assessment due to severe attrition bias.
Crotty M, Whitehead C, Rowett D, Halbert J, Weller D, Finucane P, Esterman A: **An outreach intervention to implement evidence based practice in residential care: a randomized controlled trial [ISRCTN67855475].** *BMC Health Serv Res* 2004, **4**:6.	Intervention only partially guideline based, guideline not available. Both high-level (nursing homes) and low-level care (hostels), no separate outcomes reported.
Dharmarajan TS, Nanda A, Agarwal B, Agnihotri P, Doxsie GL, Gokula M, Javaheri A, Kanagala M, Lebelt AS, Madireddy P, Mahapatra S, Murakonda P, Muthavarapu SRR, Patel M, Patterson C, Soch K, Troncales A, Yaokim K, Kroft R, Norkus EP: **Prevention of venous thromboembolism in long term care: results of a multicenter educational intervention using clinical practice guidelines: part 2 of 2 (an AMDA Foundation project).** *J Am Med Dir Assoc* 2012, **13**:303–7.	Intervention not described.
Ersek M, Polissar N, Pen AD, Jablonski A, Herr K, Neradilek MB: **Addressing methodological challenges in implementing the nursing home pain management algorithm randomized controlled trial.** *Clin Trials Lond Engl* 2012, **9**:634–44.	Missing outcome data.
Gopal Rao G, Jeanes A, Russell H, Wilson D, Atere-Roberts E, O'Sullivan D, Donaldson N: **Effectiveness of short-term, enhanced, infection control support in improving compliance with infection control guidelines and practice in nursing homes: a cluster randomized trial.** *Epidemiol Infect* 2009, **137**:1465–71.	Guidelines used not stated and not in reference list.
Gotoh M, Yoshikawa Y, Ono Y, Ohshima S: **Effectiveness of the introduction of a guideline for urinary management in the elderly at nursing homes**. *J Urol* 2005, **173**:4–4.	Article unable to be retrieved.
Hutt E, Ruscin JM, Corbett K, Radcliff TA, Kramer AM, Williams EM, Liebrecht D, Klenke W, Hartmann S: **A multifaceted intervention to implement guidelines improved treatment of nursing home-acquired pneumonia in a state veterans home.** *J Am Geriatr Soc* 2006, **54**:1694–700.	Guideline not based on a review of the literature.

Study reference	Reason for exclusion
Hutt E, Ruscin JM, Linnebur SA, Fish DN, Oman KS, Fink RM, Radcliff TA, Van Dorsten B, Liebrecht D, Fish R, McNulty MC: **A multifaceted intervention to implement guidelines did not affect hospitalization rates for nursing home-acquired pneumonia.** *J Am Med Dir Assoc* 2011, **12**:499–507.	Guideline not based on a review of the literature.
Jones K, Fink R, Vojir C, Pepper G, Hutt E, Clark L, Scott J, Martinez R, Vincent D, Mellis B: **Translation research in long-term care: improving pain management in nursing homes.** *Worldviews Evid Based Nurs* 2004, **1**:S13–20.	Participants healthcare personnel, family and residents. No separate analysis.
Naughton BJ, Mylotte JM, Ramadan F, Karuza J, Priore RL: **Antibiotic use, hospital admissions, and mortality before and after implementing guidelines for nursing home-acquired pneumonia.** *J Am Geriatr Soc* 2001, **49**:1020–4.	Guideline based on experience and community practice. No review of the literature.
Rapp MA, Mell T, Majic T, Treusch Y, Nordheim J, Niemann-Mirmehdi M, Gutzmann H, Heinz A: **Agitation in nursing home residents with dementia (VIDEANT trial): effects of a cluster-randomized, controlled, guideline implementation trial.** *J Am Med Dir Assoc* 2013, **14**:690–5.	Consensus guideline, not evidence-based.
Schmidt I, Claesson CB, Westerholm B, Nilsson LG, Svarstad BL: **The impact of regular multidisciplinary team interventions on psychotropic prescribing in Swedish nursing homes**. *J Am Geriatr Soc* 1998, **46**:77–82.	Not explicitely guideline-based. Guideline unable to be retrieved, not publically available.
Testad I, Aasland AM, Aarsland D: **The effect of staff training on the use of restraint in dementia: a single-blind randomised controlled trial.** *Int J Geriatr Psychiatry* 2005, **20**:587–90.	Intervention only partially guideline-based. Guideline not stated and not in reference list.
Verkaik R, Francke AL, van Meijel B, Spreeuwenberg PMM, Ribbe MW, Bensing JM: **The effects of a nursing guideline on depression in psychogeriatric nursing home residents with dementia.** *Int J Geriatr Psychiatry* 2011, **26**:723–32.	Guideline not based on a review of the literature, not based on current evidence.
Westbury J, Jackson S, Gee P, Peterson G: **An effective approach to decrease antipsychotic and benzodiazepine use in nursing homes: the RedUSe project.** *Int Psychogeriatr IPA* 2010, **22**:26–36.	Guidelines not based on a review of the literature, not based on current evidence.
Zimmerman S, Sloane PD, Bertrand R, Olsho LEW, Beeber A, Kistler C, Hadden L, Edwards A, Weber DJ, Mitchell CM: **Successfully reducing antibiotic prescribing in nursing homes.** *J Am Geriatr Soc* 2014, **62**:907–12.	Guideline not evidence-based, no review of the literature.
