# Supplementary material for: Implementing guidelines in nursing homes: a systematic review
Source: BMC Health Serv Res. 2016 Jul 25;16:298. doi: 10.1186/s12913-016-1550-z (PMC4960750; doi:10.1186/s12913-016-1550-z)
Supplement: Additional file 4: — Summary of findings tables (professional practice). Summary of findings tables for outcomes classified as professional practice. (PDF 84 kb) [file 12913_2016_1550_MOESM4_ESM.pdf]

# Additional file 4 – Summary of findings tables (professional practice)

Table S1

## A multifaceted theory-based educational intervention compared to standard information for the implementation of best practices to reduce physical restraints

**Patient or population**: Healthcare personnel
**Setting**: Nursing homes in Germany
**Intervention**: A multifaceted theory-based educational intervention
**Comparison**: Usual care

| Outcomes | **Anticipated absolute effects*** (95% CI) | | Relative effect (95% CI) | № of participants (Studies) | Quality of the evidence (GRADE) | Comments |
|---|---|---|---|---|---|---|
| | **Risk with standard information** | **Risk with a multifaceted theory-based educational intervention** | | | | |
| **Residents with physical restraints.** Assessed with: direct observation by external investigators. Follow up: 3 months | **Study population** | | **RR 0.79** (0.64 to 0.97) | 36 nursing homes, 3670 residents (1 Cluster-RCT) | ⨁⨁◯◯ LOW [1,2] | P=0.025 Results corrected for cluster. |
| | 305 per 1000 | **241 per 1000 (195 to 196)** | | | | |
| **Residents with physical restraints.** Assessed with: direct observation by external investigators Follow up: 6 months | **Study population** | | **RR 0.78** (0.63 to 0.97) | 36 nursing homes, 3664 residents (1 Cluster-RCT) | ⨁⨁◯◯ LOW [1,2] | P=0.024 Results corrected for cluster. |
| | 291 per 1000 | **227 per 1000 (181 to 283)** | | | | |

1. Only one single study
2. Wide confidence interval

Table S2

## A multifaceted toolkit-based intervention compared to plain toolkit dissemination for the implementation of atypical antispychotic prescribing best practice

**Patient or population**: Healthcare personnel
**Setting**: Nursing homes in the US
**Intervention**: A multifaceted toolkit-based intervention
**Comparison**: Plain toolkit dissemination

| Outcomes | Anticipated absolute effects* (95% CI) | | Relative effect (95% CI) | № of participants (Studies) | Quality of the evidence (GRADE) | Comments |
|---|---|---|---|---|---|---|
| | **Risk with plain toolkit dissemination** | **Risk with a single toolkit-based intervention** | | | | |
| **Prevalence of atypical antipsychotic use**<br>Assessed with: screening of pharmacy dispensing data.<br>Follow up: 12 months | **Study population** | | Not estimable | 42 nursing homes, number of participating residents unknown<br>(1 Cluster-RCT) | ⨁◯◯◯<br>VERY LOW [1,2] | There was no difference in the postintervention changes in the prescribing of atypical antipsychotics (slope or level) compared to the control group. |
| | Not estimable | Not estimable | | | | |

1. External contamination in intervention and control groups, high risk of contamination bias. Unclear allocation concealment, possibility of selection bias.
2. Only one single study with few events. Study seriously underpowered.

Table S3

## Another multifaceted toolkit-based intervention compared to plain toolkit dissemination for the implementation of atypical antispychotic prescribing best practice

**Patient or population**: Healthcare personnel
**Setting**: Nursing homes in the US
**Intervention**: Another multifaceted toolkit-based intervention
**Comparison**: Plain toolkit dissemination

| Outcomes | Anticipated absolute effects* (95% CI) | | Relative effect (95% CI) | № of participants (Studies) | Quality of the evidence (GRADE) | Comments |
|---|---|---|---|---|---|---|
| | **Risk with plain toolkit dissemination** | **Risk with a single toolkit-based intervention** | | | | |
| **Prevalence of atypical antipsychotic use** Assessed with: screening of pharmacy dispensing data. Follow up: 12 months | **Study population** | | Not estimable | 42 nursing homes, number of participating residents unknown (1 Cluster-RCT) | ⊕◯◯◯ VERY LOW [1,2] | There was no difference in the postintervention changes in the prescribing of atypical antipsychotics (slope or level) compared to the control group. |
| | Not estimable | Not estimable | | | | |

1. External contamination in the intervention and control groups, high risk of contamination bias. Unclear allocation concealment, possibility of selection bias.
2. Only one single study with few events. Study seriously underpowered.

Table S4

## The patient safety programme "SAFE OR SORRY?" compared to usual care for the implementation of pressure ulcer, urinary tract infection and falls best practice guidelines

**Patient or population**: Healthcare personnel
**Setting**: Nursing homes in Netherland
**Intervention**: The patient safety programme "SAFE OR SORRY?"
**Comparison**: Usual care

| Outcomes | Anticipated absolute effects* (95% CI) | | Relative effect (95% CI) | № of participants (Studies) | Quality of the evidence (GRADE) | Comments |
|---|---|---|---|---|---|---|
| | Risk with usual care | Risk with the patient safety programme "SAFE OR SORRY?" | | | | |
| **Adequate care given to patients at risk for pressure ulcers** Assessed with: chart review and patient observation by independent research assistants. Follow up: 9 months | Study population 128 per 1000 | **204 per 1000 (120 to 351)** | **RR 1.60** (0.94 to 2.75) | 10 wards from 6 nursing homes, 392 residents (1 Cluster-RCT) | ⨁◯◯◯ VERY LOW [1,2,3,4] | P=0.084 Results corrected for cluster. |
| **Adequate care given to patients at risk for urinary tract infections.** Assessed with: chart review and patient observation by independent research assistants. Follow up: 9 months | Study population 408 per 1000 | **445 per 1000 (367 to 539)** | **RR 1.09** (0.90 to 1.32) | 10 wards from 6 nursing homes, 392 residents (1 Cluster-RCT) | ⨁◯◯◯ VERY LOW [1,2,4] | P=0.37 Results corrected for cluster. |
| **Adequate care given to patients at risk for falls.** Assessed with: chart review and patient observation by independent research assistants. Follow up: 9 months | Study population Not estimable | Not estimable | Not estimable | 10 wards from 6 nursing homes, 392 residents (1 Cluster-RCT) | ⨁◯◯◯ VERY LOW [1,2,3,4] | 1% or fewer events in both intervention and control groups. Percentages too low for statistical analysis. |

1. Participants allocated after randomization, unclear risk of selection bias. Intervention and control wards within the same nursing home, high risk of contamination bias.
2. Only one single study with few events.
3. Wide confidence interval.
4. Small sample size.

Table S5

## The employment of a project nurse compared to usual care for the implementation of falls best practice strategies

**Patient or population**: Healthcare personnel
**Setting**: Nursing homes in Australia
**Intervention**: The employment of a project nurse
**Comparison**: Usual care

| Outcomes | Anticipated absolute effects* (95% CI) | | Relative effect (95% CI) | № of participants (Studies) | Quality of the evidence (GRADE) | Comments |
|---|---|---|---|---|---|---|
| | **Risk with usual care** | **Risk with the employment of a project nurse** | | | | |
| **The use of vitamin D supplements** Assessed with: Monthly chart review by the nursing home staff. Follow up: 17 months | **Study population** | | Not estimable | 88 nursing homes, 5391 residents (1 Cluster-RCT) | ⨁◯◯◯ VERY LOW [1,2,3] | Increase in the use of vitamin D supplements with mean slope of 2.0 supplements per 100 beds per month (P < 0.001) averaged over both groups. No difference between intervention and control group (P = 0.092). No confidence interval supplied. Results corrected for cluster. |
| | Not estimable | Not estimable | | | | |
| **The use of hip protectors** Assessed with: Monthly chart review by the nursing home staff. Follow up: 17 months | **Study population** | | Not estimable | 88 nursing homes, 5391 residents (1 Cluster-RCT) | ⨁◯◯◯ VERY LOW [1,2,3] | Small increase in the use of hip protectors in both groups: 0.29 per 100 beds per month (95% CI, 0.17 to 0.41; P < 0.001). No difference between intervention and control group (P > 0.05). Results corrected for cluster. |
| | Not estimable | Not estimable | | | | |

1. Contamination between intervention and control group, high risk of contamination bias. Unclear allocation concealment, possibility of selection bias. Self-reporting, high risk of detection bias.
2. Only one single study with few events.
3. Large confidence interval.
