# Supplementary material for: Implementing guidelines in nursing homes: a systematic review
Source: BMC Health Serv Res. 2016 Jul 25;16:298. doi: 10.1186/s12913-016-1550-z (PMC4960750; doi:10.1186/s12913-016-1550-z)
Supplement: Additional file 5: — Summary of findings tables (patient outcome). Summary of findings tables for outcomes classified as patient outcome. (PDF 77 kb) [file 12913_2016_1550_MOESM5_ESM.pdf]

# Additional file 5 – Summary of findings tables (patient outcome)

Table S6

## Supervision by an oral health care team compared to guideline dissemination for the implementation of an oral health care guideline

**Patient or population**: Healthcare personnel
**Setting**: Nursing homes in Belgium
**Intervention**: Supervision by an oral health care team
**Comparison**: Guideline dissemination

| Outcomes | Anticipated absolute effects* (95% CI) | | Relative effect (95% CI) | № of participants (Studies) | Quality of the evidence (GRADE) | Comments |
|---|---|---|---|---|---|---|
| | **Risk with guideline dissemination** | **Risk with supervision by an oral health care team** | | | | |
| **Tongue plaque level** Assessed with: Oral examination by external investigators. Scale from: 0 to 12 Follow up: 6 months | The mean tongue plaque level in the control group was **3.66** plaque index score points. | The mean tongue plaque level in the intervention group was 0.07 lower (0.91 lower to 0.77 higher) | Not estimable. | 12 nursing homes, 278 residents (1 Cluster-RCT) | ⊕⊕◯◯ LOW [1 2] | P = 0.87 Results corrected for cluster and baseline differences. |
| **Dental plaque level.** Assessed with: Oral examination by external investigators. Scale from: 0 to 3 Follow up: 6 months | The mean dental plaque level. in the control group was **1.77** plaque index score points. | The mean dental plaque level. in the intervention group was 0.15 lower (0.45 lower to 0.14 higher) | Not estimable. | 12 nursing homes, 97 residents (1 Cluster-RCT) | ⊕⊕◯◯ LOW [1 2] | P = 0.32 Results corrected for cluster and baseline differences. |
| **Denture plaque level.** Assessed with: Oral examination by external investigators. Scale from: 0 to 4 Follow up: 6 months | The mean denture plaque level. in the control group was **2.37** plaque index score points. | The mean denture plaque level. in the intervention group was 0.32 lower (0.52 lower to 0.11 lower) | Not estimable. | 12 nursing homes, 194 residents (1 Cluster-RCT) | ⊕⊕◯◯ LOW [1 2] | P = 0.02 Results corrected for cluster and baseline differences. |

1. Only one single study with few events
2. Small sample size

Table S7

## The patient safety programme "SAFE OR SORRY?" compared to usual care for the implementation of pressure ulcer, urinary tract infection and falls best practice guidelines

**Patient or population**: Healthcare personnel
**Setting**: Nursing homes in Netherland
**Intervention**: The patient safety programme "SAFE OR SORRY?"
**Comparison**: Usual care

| Outcomes | Anticipated absolute effects* (95% CI) | | Relative effect (95% CI) | № of participants (Studies) | Quality of the evidence (GRADE) | Comments |
|---|---|---|---|---|---|---|
| | Risk with usual care | Risk with the patient safety programme "SAFE OR SORRY?" | | | | |
| **Incidence of adverse events (pressure ulcer, urinary tract infections and falls).** Assessed with: chart review and skin inspection by independent research assistants. Follow up: 9 months | **Study population** | | **Rate ratio 0.67** (0.47 to 0.97) | 10 wards from 6 nursing homes, 392 residents (1 Cluster-RCT) | ⊕◯◯◯ VERY LOW [1 2 3 4] | P<0.05 Intervention group 174/2754, control group 272/3045 adverse events/patient weeks. Results corrected for cluster. |
| | Mean 0.07 events/patient week | **Mean 0.07 events/patient week** | | | | |

1. Participants allocated after randomization, unclear risk of selection bias. Intervention and control wards within the same nursing home, high risk of contamination bias.
2. Only one single study with few events.
3. Wide confidence interval.
4. Small sample size.

Table S8

## The employment of a project nurse compared to usual care for the implementation of falls best practice strategies

**Patient or population**: Healthcare personnel
**Setting**: Nursing homes in Australia
**Intervention**: The employment of a project nurse
**Comparison**: Usual care

| Outcomes | **Anticipated absolute effects*** (95% CI) | | Relative effect (95% CI) | № of participants (Studies) | Quality of the evidence (GRADE) | Comments |
|---|---|---|---|---|---|---|
| | **Risk with usual care** | **Risk with the employment of a project nurse** | | | | |
| **Residents with at minimum one femoral neck fracture** Assessed with: Monthly chart review by the nursing home staff. Follow up: 17 months | **Study population** | | **RR 0.95** (0.63 to 1.43) | 88 nursing homes, 5391 residents (1 Cluster-RCT) | ⨁◯◯◯ VERY LOW [1 2 3] | P=0.79 Results corrected for cluster. |
| | 41 per 1000 | **39 per 1000 (26 to 59)** | | | | |

1. Contamination between intervention and control group, high risk of contamination bias. Unclear allocation concealment, possibility of selection bias. Self-reporting, high risk of detection bias.
2. Only one single study with few events.
3. Large confidence interval.
