# Supplementary material for: Implementing guidelines in nursing homes: a systematic review
Source: BMC Health Serv Res. 2016 Jul 25;16:298. doi: 10.1186/s12913-016-1550-z (PMC4960750; doi:10.1186/s12913-016-1550-z)
Supplement: Additional file 6: — Risk of bias assessment. A detailed description of the risk of bias assessment of all included studies. (PDF 138 kb) [file 12913_2016_1550_MOESM6_ESM.pdf]

Additional file 6 – Risk of bias assessment

**Study:** Colon-Emeric et al., 2007<br>**Design:** Cluster-randomised controlled trial		
**Domain**	**Evaluation**	**Comments**
Was the allocation sequence adequately generated?	Low risk	Quote:"*The nursing homes were randomized within each state... using a random number generator.*"
Was the allocation adequately concealed?	Low risk	Allocation by institution, performed on start of the study.
Were baseline outcome measurements similar?	High risk	Described in Table 2. Significantly higher prescription rate of vitamin D in the intervention group. Analysis not corrected. Prescription rates of Calcium and vitamin D ~ 70% in both groups. Ceiling effect.
Were baseline characteristics similar?	Low risk	Quote: "*Intervention residents were more likely to be African American, younger, and used tobacco; and less likely to have previous fracture or dysphagia.*"<br>Quote: "*..adjusting for baseline factors that were imbalanced, including bed size, age, race, sex, previous fracture, insurance status, ambulatory status, gastrointestinal reflux, breast and endometrial cancer, dysphagia, and tobacco use.*<br>Comment: imbalance at baseline statistically corrected for.
Were incomplete outcome data adequately addressed?	High risk[1]	Quote: "*Participation in the intervention activities was low..*"<br>Comment: 64-89% non-compliance in the intervention group (Table 3). Intention-to-treat not sufficient to correct for non-compliance this big. Groups no longer comparable.<br>Quote: "*All randomized facilities were analysed regardless of their participation in the study.*" Not stated if all nursing homes delivered data or if and how many were lost to-follow-up. Unclear if the authors performed intention-to-treat analysis.
Was knowledge of the allocated interventions adequately prevented during the study?	Low risk	Quote: "*Trained data collectors, blinded to intervention status, abstracted data from the medical record before and after the intervention.*"

1 Study excluded because of severe attrition bias.

Domain	Evaluation	Comments
Was the study adequately protected against contamination?	Low risk	Quote: "*Cluster-randomized, single-blind, controlled trial of a multi-modal quality improvement intervention.*" Unlikely that the control group received the intervention.
Was the study free from selective outcome reporting?	Low risk	All outcomes from the methods section reported in Table 2.
Was the study free from other risks of bias?	Low risk	Quote: "*Analysis was at the facility-level and Generalized Estimating Equation modelling was used to account for clustering.*
**Overall risk of bias: Study excluded**		

**Study:** De Visschere et al. (2012)<br>**Design:** Cluster-randomised controlled trial		
**Domain**	**Judgement**	**Support for judgement**
Was the allocation sequence adequately generated?	Unclear	Stratified cluster sampling with random allocation. No random component mentioned.
Was the allocation adequately concealed?	Low risk	Quote: "*A random sample of 12 nursing homes was randomly allocated to the intervention or the control group.*"<br>Comment: Allocation by institution and performed at the start of the study.
Were baseline outcome measurements similar?	Low risk	Quote: *"Baseline plaque levels similar in both groups. The outcome variables, tongue plaque, dental plaque and denture plaque were skewed both at baseline (T0) and at 6-month follow-up (T1). These differences have been adjusted for the corresponding baseline value of the variable as a covariate and the random effect of the institution."*
Were baseline characteristics similar?	Low risk	No significant difference in age, care-dependency, MMSE[2], co-morbidity, dental status and oral hygiene status. P = 0.05 for gender.
Were incomplete outcome data adequately addressed?	Low risk	Quote: *"No other differences were found between residents who completed the study and those who did not, indicating no evidence for a loss to follow-up effect."*<br>Comment: All wards of the respective nursing homes involved.
Was knowledge of the allocated interventions adequately prevented during the study?	Low risk	Quote: *"The primary outcome variable was the oral hygiene level of the participating residents."*<br>Quote: *"The examiners were masked."*
Was the study adequately protected against contamination?	Low risk	Allocation by institution. Unlikely that the control group received the intervention.
Was the study free from selective outcome reporting?	Low risk	All outcome measures are reported (tongue plaque, dental plaque, denture plaque).
Was the study free from other risks of bias?	Low risk	Accounted for clustering in the power calculation and data analysis.
**Overall risk of bias: Low**		

2 Mini-mental state examination.

**Study:** Köpke et al., 2012<br>**Design:** Cluster-randomised controlled trial		
**Domain**	**Judgement**	**Support for judgement**
Was the allocation sequence adequately generated?	Low risk	Quote: *"Computer-generated randomization lists were used for allocation of clusters in blocks of 4, 6, and 8 nursing homes."*
Was the allocation adequately concealed?	Low risk	Quote: *"Cluster randomized controlled trial. Allocation of clusters was performed by an external person not involved in the study."*<br>Comment: Allocation blinded and by institution. All units allocated at the start of the study. Newly admitted residents were included after randomisation into the group the respective nursing home was assigned to and uninfluenced by the investigators. Therefore low risk of selection bias.
Were baseline outcome measurements similar?	Unclear risk	Residents with physical restraints / restraint use: Table 2. Psychotropic medicine prescriptions: Table 4. Falls and fall-related fractures: Table 1 (Characteristics!). Most probably no important differences. However, p-values are missing.
Were baseline characteristics similar?	Low risk	Stated in Table 1, similar.
Were incomplete outcome data adequately addressed?	Low risk	Quote: *"Analyses were by intention to treat; no participants or clusters changed groups and no cluster dropped out during follow-up."*<br>Comment: However, there was drop-out of individual participants, which was distributed similar between both groups. All drop-outs due to death or movement.
Was knowledge of the allocated interventions adequately prevented during the study?	Low risk	Quote: "*Statistical analyses were conducted after the end of follow-up by the statistician (B.H.), who was unaware of group allocation of clusters."*<br>Quote: *"Data on prevalence of physical restraint use at the 3- and 6-month follow-ups were assessed similarly to baseline by external investigators blinded to cluster group allocation."*<br>Comment: Data collection and analysis performed by blinded investigators.

**Domain**	**Judgement**	**Support for judgement**
Was the study adequately protected against contamination?	Low risk	Quote: *"Cluster randomized controlled trial."*<br>Comment: Allocation by institution. Unlikely that the control group received the intervention.
Was the study free from selective outcome reporting?	Low risk	Results for all outcomes reported (Table 2, 3 and 4).
Was the study free from other risks of bias?	Low risk	Accounted for clustering in sample size calculation and in the data analysis.
**Overall risk of bias: Low**		

**Study:** Tjia et al., 2015<br>**Design:** Cluster-randomized controlled trial		
**Domain**	**Judgement**	**Support for judgement**
Was the allocation sequence adequately generated?	Unclear risk	Quote: *"Using a stratified randomization procedure, participating NHs were stratified into tertiles according to facility-level prevalence of atypical antipsychotic prescribing."*<br>*Quote: "Within each tertile, NHs were further categorized and assigned into blocks[...] Simple randomization was performed within each block to assign subjects to a study arm."*<br>Comment: Stratified sampling with block-randomisation. No random component stated.
Was the allocation adequately concealed?	Unclear risk	Quote: *"The study design was a three-arm, cluster-randomized trial."*<br>Comment: Allocation by institution. Not stated if participants were allocated at the start of the study or who allocated them. Staff and researchers were aware of the allocation. Concealment of allocation not stated.
Were baseline outcome measurements similar?	Low risk	Quote: *"In the 12 months before the study intervention, the average prevalence of atypical antipsychotic use was similar across the three study arms."*
Were baseline characteristics similar?	Low risk	Comment: Characteristics shown in Table 2 appear similar.
Were incomplete outcome data adequately addressed?	Low risk	Comment: No attrition reported after randomisation.
Was knowledge of the allocated interventions adequately prevented during the study?	Low risk	Quote: *"..a three-arm cluster randomized trial was conducted using incrementally more-intensive dissemination strategies that evaluated the primary outcome of facility-level change in antipsychotic prescribing."*<br>Quote: *"Pharmacy dispensing data were used for these measures.."*<br>Comment: Monthly data collection. Not stated who collected the data.The nursing home staff was aware of the allocation of the intervention. However, pharmacy dispensing data are objective.

**Domain**	**Judgement**	**Support for judgement**
Was the study adequately protected against contamination?	High risk	Quote: *"First, during the period of the study, there were compelling secular trends with the onset of the Centers for Medicare and Medicaid Services (CMS) campaign to reduce antipsychotic medications nationwide.."*<br>Quote: *"Widespread attention toward antipsychotic reductions was evidenced in numerous high-profile newspaper articles and an Office of Inspector General Report."*<br>Quote: *"..it is likely that spillovers from external efforts contributed to reductions in antipsychotic use and reduced the ability to detect an independent effect of the intervention."*<br>Comment: Most probably there was external contamination to an unknown degree.
Was the study free from selective outcome reporting?	Low risk	Antipsychotic prescription rates at 6 months not reported. However, no significant difference between the groups after 12 months.
Was the study free from other risks of bias?	High risk	Nursing home unit of analysis. Number of participating nursing homes in the different arms too low to detect anything but a huge effect.
**Overall risk of bias: High**		

**Study:** Van Gaal et al., 2011a<br>**Design:** Cluster-randomised controlled trial (PART-I)		
**Domain**	**Judgement**	**Support for judgement**
Was the allocation sequence adequately generated?	Unclear risk	Quote:*"The randomisation of the wards was stratified for institute and type of ward and each ward was considered as a cluster. The ten hospital wards and ten nursing home wards were assigned to an intervention or usual care group."*<br>Comment: No random component mentioned.
Was the allocation adequately concealed?	Unclear risk	Quote: "*The ten hospital wards and ten nursing home wards were assigned to an intervention or usual care group."*<br>Comment: Unit of allocation by team.<br>Quote: *"Nursing home patients were asked to participate at the start of the data collection periods, or within two weeks after admission."*<br>Quote: *"Although we included the majority of the patients admitted, it is possible that this caused some minor selection bias."*<br>Comment: Participants allocated after randomisation. Not stated who allocated them. Staff and researchers were aware of the allocation.
Were baseline outcome measurements similar?	Low risk	Quote: "*After the randomisation, baseline data were collected during three months at all wards, followed by the implementation of the patient safety programme in the intervention group."*<br>Comment: Baseline outcomes measured prior to the intervention.<br>Quote: *"Results (are) rate ratio from a Poisson regression model using ward as random factor the offset was the duration of observation and institution patients at risk for an AE[3] at the first visit and the incidence of AEs from each ward at baseline."*<br>Comment: Baseline outcome measures similar, Table 4. Adjusted for baseline differences in the analysis.

3 Adverse events (pressure ulcers, urinary tract infections, falls)

Domain	Judgement	Support for judgement
Were baseline characteristics similar?	Low risk	Quote: *"Table 3 presents the characteristics of the patients included in the intervention and usual care group at baseline and at follow-up."*<br>Comment: Nearly half as much physically impaired residents and twice as much rehabilitation residents in the intervention group. Table 1: more wards with physically impaired residents in the intervention group, and more rehabilitation wards in the control group. Number of residents at risk for adverse events and falls similar.<br>Quote: "*...analysed using a random effects Poisson regression analysis, including the following covariates: ward (random effect), institution and the baseline results of the ward."*<br>Comment: Corrected for baseline imbalance in the data analysis.
Were incomplete outcome data adequately addressed?	High risk	Quote: *"Analyses were performed by intention to treat."*<br>Comment: loss to follow-up 20% in the intervention and 31% in the control group (refused with cause unknown, discharged or died). Analysed by intention to treat.
Was knowledge of the allocated interventions adequately prevented during the study?	Low risk	Quote: *"To ensure the validity of the results, all data were collected by independent research assistants who were trained in reading patient files..."*<br>Quote: *"Trained independent research assistants collected the data in: (1) a weekly visit, and (2) by three additional observations on every ward."*<br>Comment: Investigators collecting data were unaware of allocation.
Was the study adequately protected against contamination?	High risk	Quote: *"Cluster randomized controlled trial .."*<br>Comment: Allocation by institution.<br>Quote (design): *"The randomisation of the wards was stratified for centre and type of ward (Figure 1).."*<br>Comment: 6 nursing homes with a total of 10 wards participated. Impossible that the nursing home(s) with more than one participating ward only hosted wards within the same group. Contamination likely.

Domain	Judgement	Support for judgement
Was the study free from selective outcome reporting?	Low risk	All relevant outcomes in the methods section are reported in the results section.
Was the study free from other risks of bias?	Low risk	Quote (design): *"As randomisation was on ward level, a ward was considered to be a cluster. To account for these clusters an intra class correlation coefficient of 0.01 was used in the calculation."*<br>Comments: results corrected for clustering.
**Overall risk of bias: High**		

**Study:** Van Gaal et al., 2011b<br>**Design:** Cluster-randomized controlled trial (PART-II)[4]		
**Domain**	**Judgement**	**Support for judgement**
Was the allocation sequence adequately generated?	Unclear	Quote: *"As described in Part I, ten wards from four hospitals and ten wards from six nursing homes were stratified for institute and ward type and then randomised to intervention or usual care group."*<br>Comment: No random component mentioned.
Was the allocation adequately concealed?	Unclear	Quote: "*As described in Part I, ten wards from four hospitals and ten wards from six nursing homes were stratified for institute and ward type and then randomised to intervention or usual care group."*<br>Comment: Unit of allocation by team.<br>Quote: *"Nursing home patients were asked to participate at the start of the data collection periods, or within two weeks after admission."*<br>Comment: Participants allocated after randomisation. Not stated who allocated them. Staff and investigators were aware of the allocation.
Were baseline outcome measurements similar?	Low risk	Quote (Part I): "*After the randomisation, baseline data were collected during three months at all wards, followed by the implementation of the patient safety programme in the intervention group."*<br>Comment: Baseline outcomes measured prior to the intervention.<br>Quote: *"The results of this study were clustered to ward level, so we used random effects analyses with ward as random factor. Group, institution and the baseline results of the ward were fixed covariates."*<br>Comment: Baseline outcome measures slightly different for all of the three main outcome measures, Table 3. Adjusted for in the analysis.

4*"The design and setting of the cluster randomised trial, which was conducted between September 2006 and November 2008, has been described in Part I."* (Van Gaal et al., 2011b).

Domain	Judgement	Support for judgement
Were baseline characteristics similar?	Low risk	Quote: *"The characteristics of the patients included in the intervention and the usual care group at baseline and follow-up have been described in Part I of this study".* Quote (Part I): *"Table 3 presents the characteristics of the patients included in the intervention and usual care group at baseline and at follow-up."* Comment: Nearly half as much physically impaired residents and twice as much rehabilitation residents in the intervention group. Number of residents at risk for adverse events similar. Quote: *"The results of this study were clustered to ward level, so we used random effects analyses with ward as random factor. Group, institution and the baseline results of the ward were fixed covariates."* Comment: Corrected for baseline imbalance in the data analysis.
Were incomplete outcome data adequately addressed?	High risk	Quote (Part I): *"Analyses were performed by intention to treat."* Comment: loss to follow-up 20% in the intervention and 31% in the control group (refused, discharged or died). Analysis by intention to treat insufficient.
Was knowledge of the allocated interventions adequately prevented during the study?	Low risk	Quote: *Trained independent research assistants collected the data in: (1) a weekly visit, and (2) by three additional observations on every ward."* Comment: Investigators collecting data were unaware of allocation.
Was the study adequately protected against contamination?	High risk	Quote: *"Cluster randomized controlled trial .."* Comment: Allocation by institution. Quote (design): *"The randomisation of the wards was stratified for centre and type of ward (Figure 1).."* Comment: 6 nursing homes with a total of 10 wards participated. Mathematically impossible that the nursing home(s) hosting more than one participating ward only hosted wards within the same group. Contamination likely.
Was the study free from selective outcome reporting?	Low risk	All relevant outcomes in the methods section reported in the results section.

**Domain**	**Judgement**	**Support for judgement**
Was the study free from other risks of bias?	Low risk	Quote (design): *"As randomisation was on ward level, a ward was considered to be a cluster. To account for these clusters an intra class correlation coefficient of 0.01 was used in the calculation."*<br>Comment: results corrected for clustering.
**Overall risk of bias: High risk**		

**Study:** Ward et al., 2010<br>**Design:** Cluster-randomized controlled trial		
**Domain**	**Judgement**	**Support for judgement**
Was the allocation sequence adequately generated?	Low risk	Quote: *"Consenting facilities were.. randomly allocated within strata into intervention or control groups by the statistician (R E G) using the procedure "surveyselect" in SAS statistical software."*
Was the allocation adequately concealed?	Unclear risk	Quote: *"We undertook a cluster randomised controlled trial."*<br>Comment: Allocation by institution. Not stated if participants were allocated at the start of the study or who allocated them. Staff and researchers were aware of the allocation.
Were baseline outcome measurements similar?	Low risk	Quote: *"Mean use of vitamin D at baseline was 12.7 supplements per 100 beds (95% CI, 7.4 to 18.1) in the control group and was 6.7 per 100 beds (95% CI, 1.2 to 10.9) lower in the intervention group. However, there were no differences in slopes, for either the first or second stage..with respect to study group."*<br>Comment: No differences between study groups. Therefore unlikely that the results are biased. Baseline outcome measurements similar for the use of hip protectors and fall rates.
Were baseline characteristics similar?	Low risk	Quote: *"Box 1 shows that randomisation produced reasonably similar characteristics for residents in the control and intervention groups. Consenting facilities were stratified."*
Were incomplete outcome data adequately addressed?	Low risk	Quote: *"Overall, six facilities withdrew from the project during the intervention. All withdrawing facilities provided sufficient data to allow retention in analyses. All facilities were analysed according to random allocation (intention to treat)."*

**Domain**	**Judgement**	**Support for judgement**
Was knowledge of the allocated interventions adequately prevented during the study?	High risk	Quote: *"The main outcomes of interest were change in use of vitamin D supplements and hip protectors, and change in the rate of fall events."*<br>Comment: Monthly data collection/reporting on falls, vitamin D supplements and the use of hip protectors by the nursing home staff (self-reporting), who were aware of the allocation of the intervention.<br>*Quote: "Failure to produce monthly data was followed up by the project nurse."*<br>Comment: The project nurse was aware of the allocation.
Was the study adequately protected against contamination?	High risk	Quote: *"There was also a possibility of contamination between the intervention and control groups with regard to the introduction of the strategies. This almost certainly happened, because falls prevention was promoted widely by NSW Health to aged care facilities during this period. In addition, doctors responsible for prescription of calcium and vitamin D supplements visited both the intervention and control facilities."*<br>Comment: The physicians could also have introduced (parts of) the intervention to the control group.
Was the study free from selective outcome reporting?	Low risk	All outcomes from the methods section reported in the results section.
Was the study free from other risks of bias?	Unclear risk	Results cluster-corrected, but most probably not for main outcome "Residents with at minimum one femoral neck fracture".
**Overall risk of bias: High**		
